# Supplementary figures and images for: Adaptations of the axon initial segment in fast-spiking interneurons of the human neocortex support low action potential thresholds
Source: PLoS Biol. 2025 Dec 10;23(12):e3003549. doi: 10.1371/journal.pbio.3003549 (PMC12798858; doi:10.1371/journal.pbio.3003549)

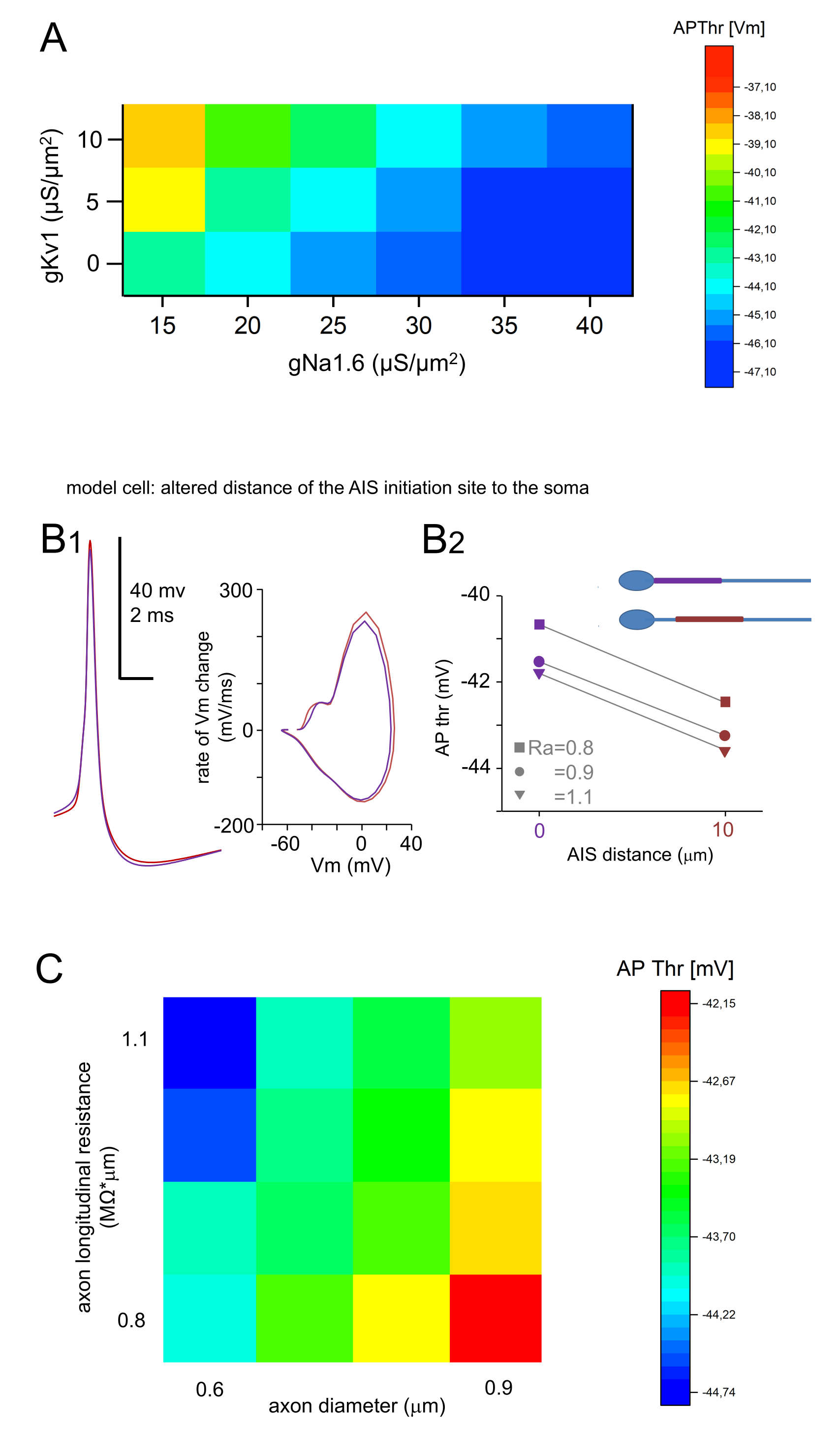

Supplement: S1 Fig — (A) Increasing AIS Nav1.6 conductance (gNav1.6, 15–40 nS/µm2; abscissa) progressively lowered the AP firing threshold across multiple Kv1 activity levels (0–10 nS/µm2). AIS length = 20 µm; axon Ra = 0.8–1.1 MΩ·µm; axon diameter = 0.8 µm. (B) Proximal–distal positioning of the AIS regulated the AP firing threshold within the range observed anatomically. Simulations used square-pulse current steps to mimic Vm changes (as shown in Fig 8). (B1) Vm traces from simulations in the model cell with the AIS positioned either directly at the axon hillock (0 µm; purple) or 10 µm distally (brown), with consistent AIS length (20 µm), axon Ra (0.8–1.1 MΩ·µm), axon diameter (0.8 µm), and channel conductances (20 nS/µm2 for Nav1.6 and Kv1). (B2) Firing threshold values plotted for AIS locations and across three Ra conditions (0.8–1.1 MΩ·µm, indicated by symbols). A more distal AIS position consistently resulted in a lower AP firing threshold, replicating the real-neuron correlation observed in Fig 3C1. (C) Heatmap shows the relationship between axon longitudinal conductance (normally reduced by axon diameter increase) and axon diameter simulating a proportional (1.3-fold) increase in both Kv1 and Nav1.6 ion conductance with increasing axon diameter, uncoupling parameters that are typically correlated in real neurons. Increasing only the Ra lowered the AP firing threshold. Conversely, increasing the ion channel conductance with an increased AIS membrane area, while keeping the channel density fixed at 20 nS/µm² for Nav1.6 and Kv1, increased the firing threshold in the model cell. This indicates the strong effect of Kv1 channels on the threshold. (TIF) [file pbio.3003549.s001.tif]
