# Supplementary material for: Adaptations of the axon initial segment in fast-spiking interneurons of the human neocortex support low action potential thresholds
Source: PLoS Biol. 2025 Dec 10;23(12):e3003549. doi: 10.1371/journal.pbio.3003549 (PMC12798858; doi:10.1371/journal.pbio.3003549)
Supplement: S1 Table — (DOCX) [file pbio.3003549.s002.docx]

| **cell code** | **pvalb+ or mRNA** | **AP Thr (mV)** | **gender** | **age (years)** | **hemisphere** | **region** | **diagnosis** |
| --- | --- | --- | --- | --- | --- | --- | --- |
| h1 | pvalb+ | -42 | F | 69 | right | occipital | subcortical neoplasia |
| h2 | pvalb+ | -37,3 | M | 38 | right | temporal | malformation |
| h3 | pvalb+ | -46,8 | M | 38 | right | temporal | malformation |
| h4 | pvalb+ | -38,5 | M | 66 | right | temporal | tumor |
| h5 | pvalb+ | -40,1 | M | 45 | left | frontal | tumor |
| h6 | pvalb+ | -48,2 | M | 21 | right | parietal | tumor |
| h7 | pvalb+ | -39,1 | M | 21 | right | parietal | tumor |
| h8 | pvalb+ | -32,9 | M | 40 | right | frontal | tumor |
| h9 | pvalb+ | -45,6 | F | 70 | left | parietal | hydrocephalus |
| h10 | pvalb+ | -40,2 | M | 69 | right | parietal | hydrocephalus |
| h11 | pvalb+ | -36,8 | M | 58 | right | frontal | tumor |
| h12 | pvalb+ | -46,1 | M | 58 | right | frontal | tumor |
| h13 | pvalb+ | -47 | F | 66 | right | frontal | tumor |
| h14 | pvalb+ | -26,6 | F | 21 | right | frontal | hydrocephalus |
| h15 | pvalb+ | -27,5 | no info | no info | left | parietal | tumor |
| h16 | pvalb+ | -35 | F | 72 | right | temporobasal | tumor |
| h17 | pvalb+ | -41,3 | F | 72 | right | temporobasal | tumor |
| h18 | pvalb+ | -46,4 | M | 47 | left | frontotemporal | tumor |
| h19 | pvalb+ | -34,7 | M | 38 | right | temporal | malformation |
| h20 | pvalb+ | -38,8 | M | 38 | right | temporal | malformation |
| h21 | pvalb+ | -45,4 | M | 34 | left | no info | malformation |
| h22 | pvalb+ | -49,1 | M | 11 | left | temporal | malformation |
| h23 | pvalb+ | -40,5 | F | 61 | right | frontal | tumor |
| h24 | pvalb+ | -27,9 | F | 17 | right | parietal | tumor |
| h25 | pvalb+ | -31,2 | F | 65 | right | frontal | colloid cysta |
| h26 | pvalb+ | -30,6 | M | 85 | left | fronto-temporal | hydrocephalus |
| h27 | pvalb+ | -37,6 | F | 28 | right | temporal | hydrocephalus |
| h28 | pvalb+ | -37 | F | 30 | no info | no info | hydrocephalus |
| h29 | pvalb+ | -39,9 | F | 40 | right | frontal | anaplastic ependymoma |
| h30 | pvalb+ | -36,8 | F | 40 | right | frontal | anaplastic ependymoma |
| h31 | pvalb+ | -35,6 | F | 42 | left | fronto-temporal | astrocytoma |
| h32 | pvalb+ | -39 | F | 78 | right | frontal | hydrocephalus |
| h33 | pvalb+ | -41,1 | F | 68 | right | temporal | hydrocephalus |
| h34 | pvalb+ | -49 | F | 68 | right | temporal | hydrocephalus |
| h35 | pvalb+ | -38,9 | F | 19 | right | frontal | subcortical neoplasia |
| h36 | pvalb+ | -55,8 | F | 19 | right | frontal | subcortical neoplasia |
| h37 | pvalb+ | -52 | F | 50 | left | occipital | astrocytoma |
| h38 | pvalb+ | -20,1 | F | 54 | right | frontal | aneurysm |
| h39 | pvalb+ | -53,1 | F | 69 | no info | no info | hydrocephalus |
| h40 | pvalb+ | -25,5 | M | 36 | right | frontal | hydrocephalus |
| h41 | pvalb+ | -30 | M | 37 | right | parietal | tumor |
| h42 | pvalb+ | -48,7 | F | 60 | right | parietal | hydrocephalus |
| h43 | pvalb+ | -31 | M | 22 | right | frontal | hydrocephalus |
| h44 | pvalb+ | -40,7 | F | 39 | right | frontal | tumor |
| h45 | pvalb+ | -47,7 | F | 63 | right | temporal | hydrocephalus |
| h46 | pvalb+ | -52,6 | F | 68 | right | temporal | hydrocephalus |
| h47 | pvalb+ | -42,2 | F | 76 | right | frontal | Hydrocephalus |

| h48 | pvalb+ | -41,1 | F | 73 | right | frontal | biopsy (MRI: multifocal lesions) |
| --- | --- | --- | --- | --- | --- | --- | --- |
| h49 | pvalb+ | -40,9 | F | 73 | right | frontal | biopsy (MRI: multifocal lesions) |
| h50 | pvalb+ | -54 | M | 33 | right | frontal | hydrocephalus |
| h51 | pvalb+ | -39 | M | 20 | right | frontal | hydrocephalus |
| h52 | pvalb+ | -42,7 | M | 49 | right | frontal | colloid cyst |
| h53 | pvalb+ | -47,6 | M | 66 | right | frontal | astrocytoma |
| h54 | pvalb+ | -36,2 | M | 67 | right | occipital | subcortical neoplasia |
| h55 | pvalb+ | -53,3 | M | 68 | no info | temporal | hydrocephalus |
| h56 | pvalb+ | -45,5 | M | 51 | no info | no info | hydrocephalus |
| h57 | pvalb+ | -38,4 | F | 47 | right | frontal | colloid cyst |
| h58 | pvalb+ | -38,4 | F | 53 | left | no info | tumor |
| h59 | pvalb+ | -45,9 | F | 30 | right | frontal | tumor |
| h60 | pvalb+ | -36,4 | F | 30 | right | frontal | tumor |
| h61 | pvalb+ | -34,8 | M | 41 | right | parietal | shunt |
| h62 | pvalb+ | -32,3 | M | 20 | right | temporal | tumor |
| h63 | pvalb+ | -37,7 | M | 43 | right | frontal | tumor |
| h64 | pvalb+ | -37 | M | 65 | right | temporal | tumor |
| h65 | pvalb+ | -35 | M | 65 | right | temporal | tumor |
| h66 | pvalb+ | -38,5 | F | 67 | right | parietal | shunt |
| h67 | pvalb+ | -40,6 | F | 55 | left | temporal | tumor |
| h68 | pvalb+ | -39,3 | F | 79 | right | parietal | hydrocephalus |
| h69 | mRNA | -31,9 | F | 51 | right | Insula | tumor |
| h70 | mRNA | -25,5 | M | no info | right | frontal | tumor |
| h71 | mRNA | -42,8 | M | no info | right | frontal | tumor |
| h72 | mRNA | -28,9 | M | no info | right | frontal | tumor |
| h73 | mRNA | -26,6 | M | no info | right | frontal | tumor |
| h74 | mRNA | -21,9 | M | no info | right | frontal | tumor |
| h75 | mRNA | -32,8 | M | 70 | left | temporal | cavernoma |
| h76 | mRNA | -37,6 | F | 51 | right | Insula | tumor |
| h77 | mRNA | -31,1 | F | 51 | right | insula | tumor |
| h78 | mRNA | -33,9 | F | 51 | right | insula | tumor |
| h79 | mRNA | -40,5 | M | no info | left | frontal | hydrocephalus |
| h80 | mRNA | -32,8 | M | 59 | right | frontal | tumor |
| h81 | pvalb+ | -19,1 | F | 46 | right | frontal | tumor |
| h82 | pvalb+ | -18,3 | F | 46 | right | frontal | tumor |
| h83 | pvalb+ | -26,1 | F | 46 | right | frontal | tumor |
| h84 | pvalb+ | -34,9 | M | 68 | left | occipital | tumor |
| h85 | pvalb+ | -43,3 | M | 68 | left | occipital | tumor |
| h86 | pvalb+ | -41,9 | M | 68 | left | occipital | tumor |
| h87 | pvalb+ | -43,3 | F | 31 | left | temporal | tumor |
| h88 | pvalb+ | -37,3 | M | 70 | right | temporal | tumor |
| h89 | pvalb+ | -40,4 | M | 70 | right | temporal | tumor |
| h90 | pvalb+ | -34,2 | M | 70 | right | temporal | tumor |
| h91 | pvalb+ | -38,2 | M | 70 | right | temporal | Tumor |
