# Supplementary material for: Adaptations of the axon initial segment in fast-spiking interneurons of the human neocortex support low action potential thresholds
Source: PLoS Biol. 2025 Dec 10;23(12):e3003549. doi: 10.1371/journal.pbio.3003549 (PMC12798858; doi:10.1371/journal.pbio.3003549)

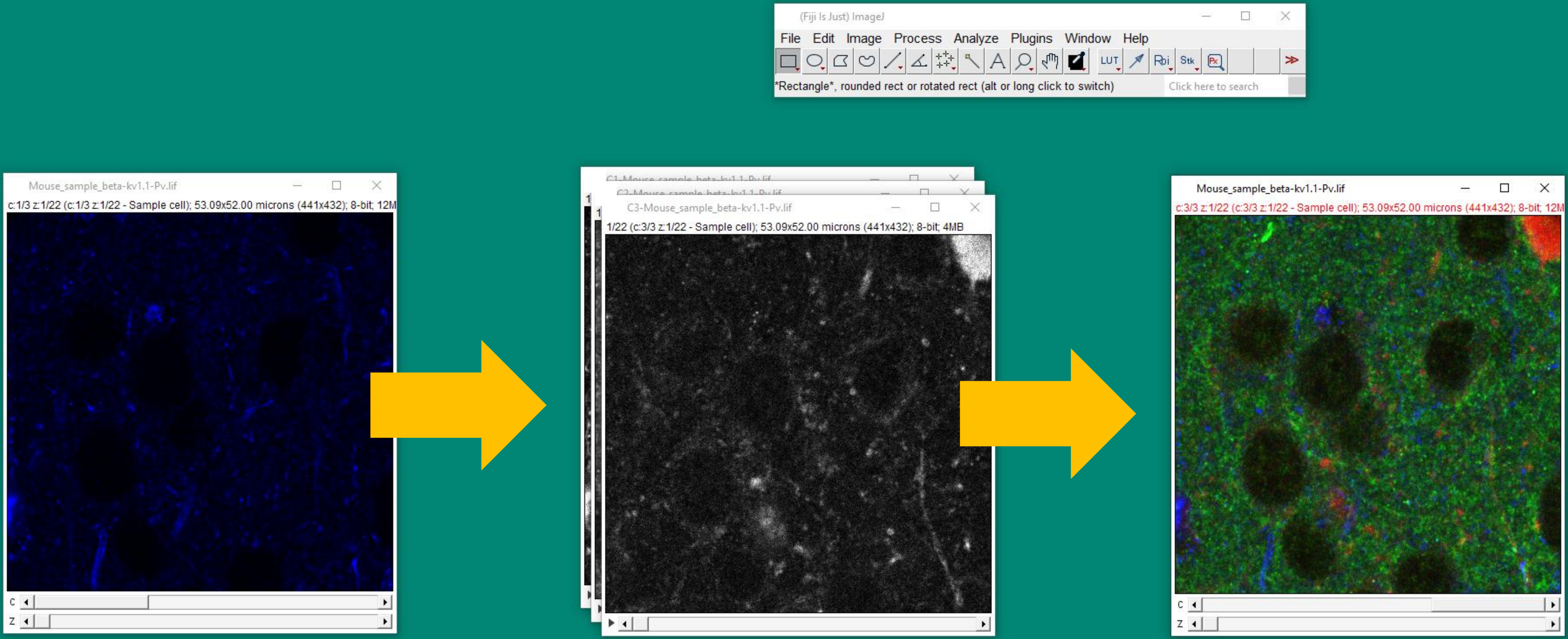

For line analysis, open image file in ImageJ
Split and merge channels to make an overlapped image series.

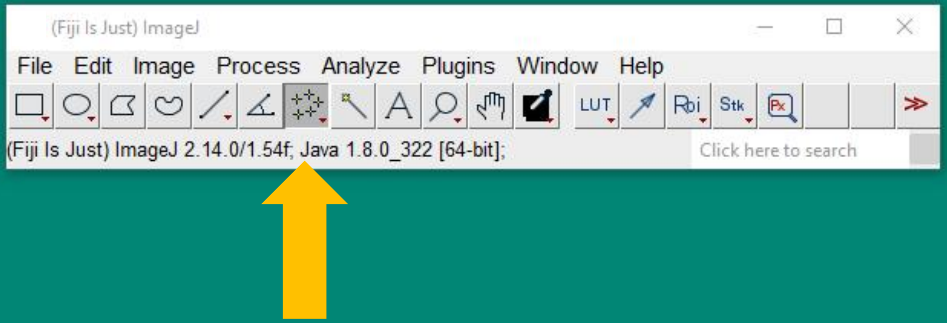

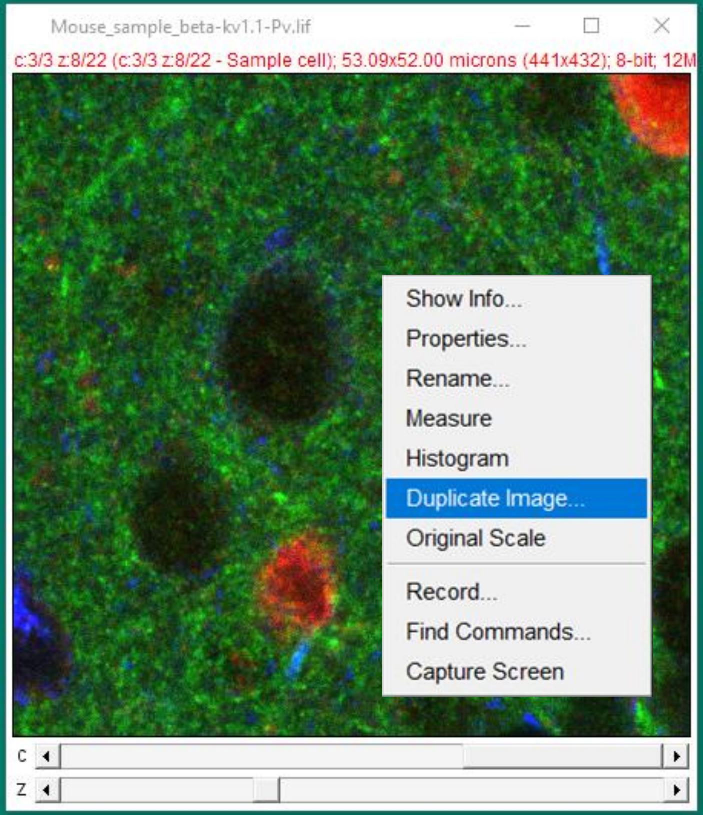

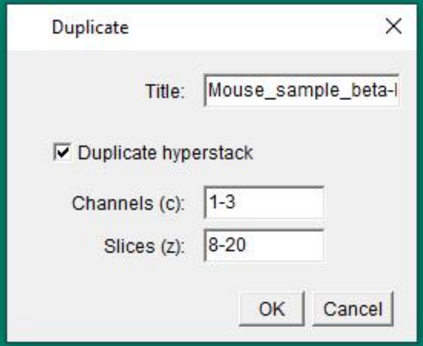

Select slices of interest and multi point tool.
Open S6_Code_ImageJ.jim

Mark roi through Z-stack. Points of beginning and end of AIS should be noted. Points outside of this range is used for background normalization. Run S6_Code_ImageJ.ijm

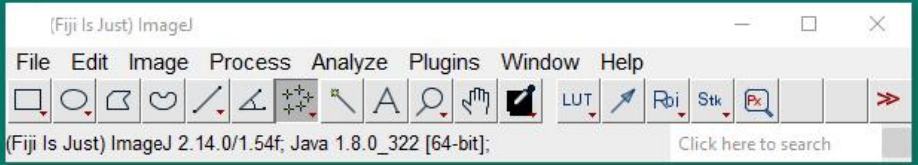

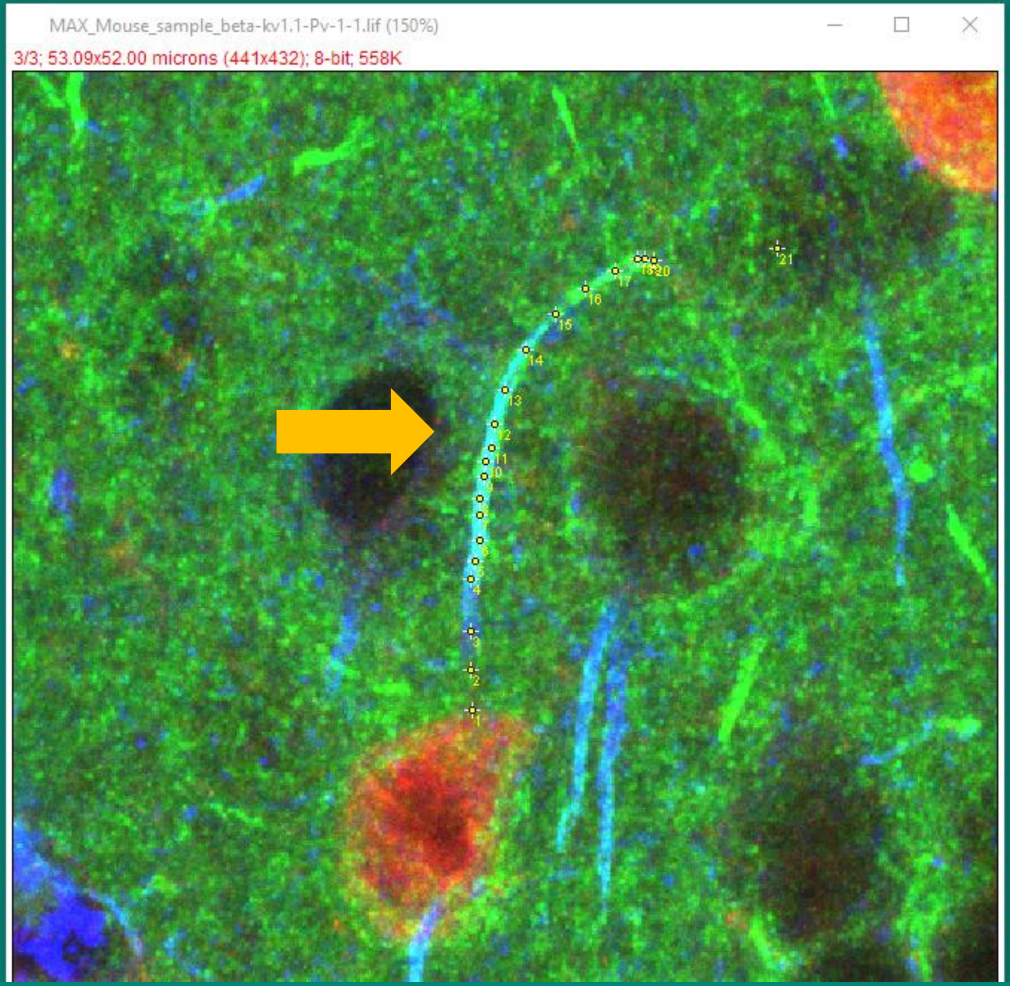

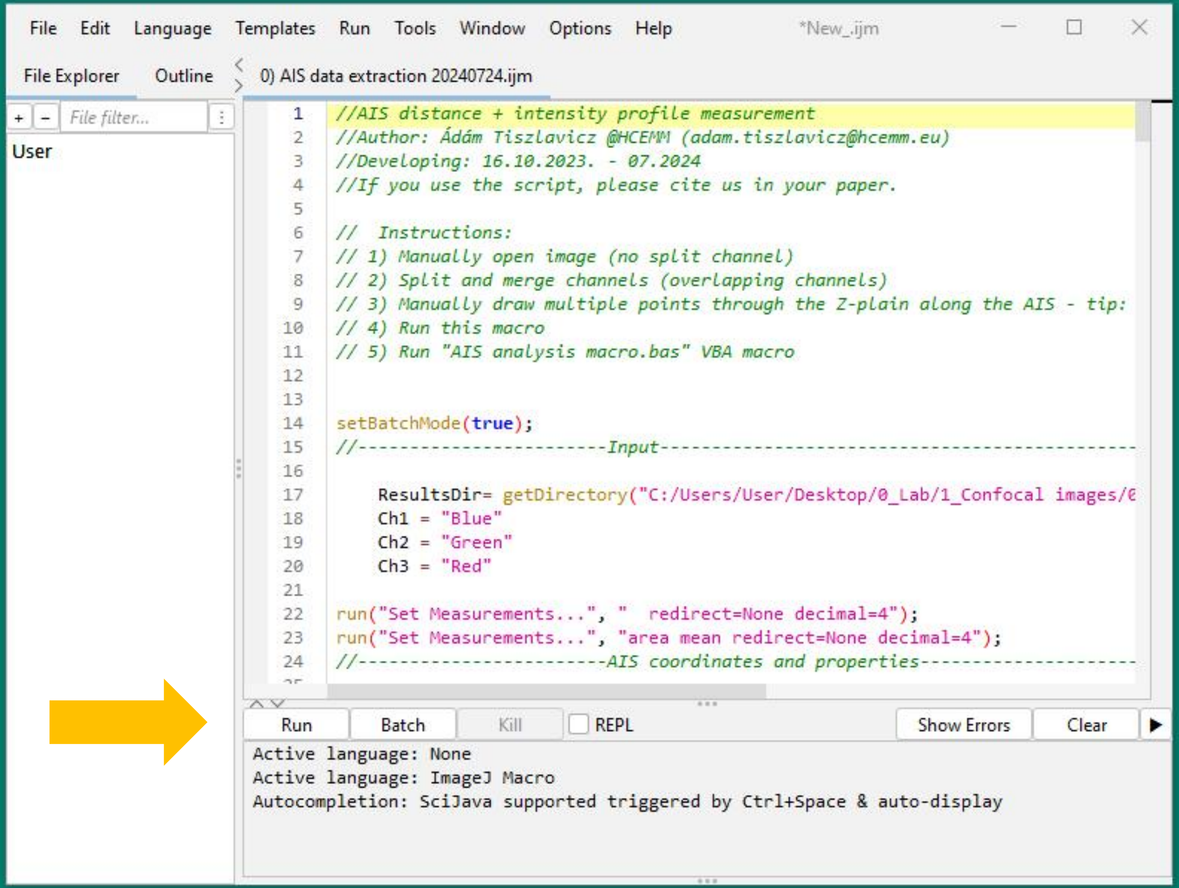

Then, the script asks for a folder in which the files of the target cell is saved.

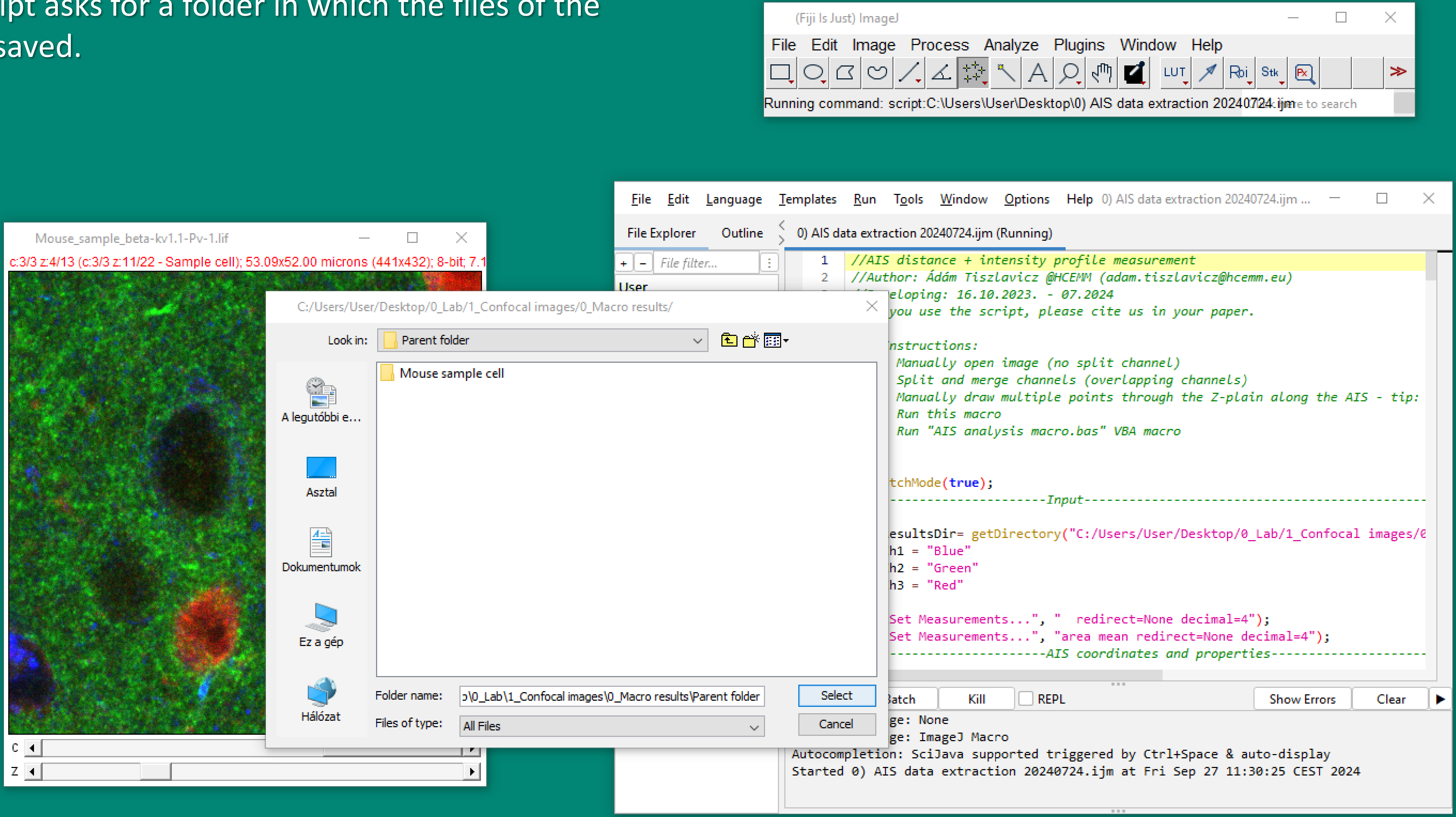

Then, parameters to be entered:
The start and end slice number of the Z-stack and mark number of the beginning and end of AIS.

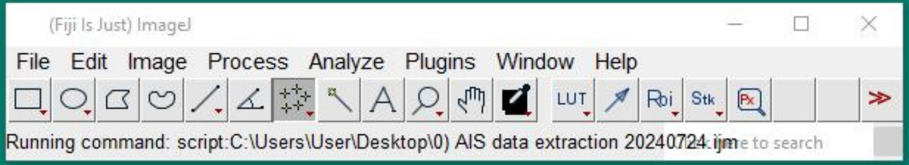

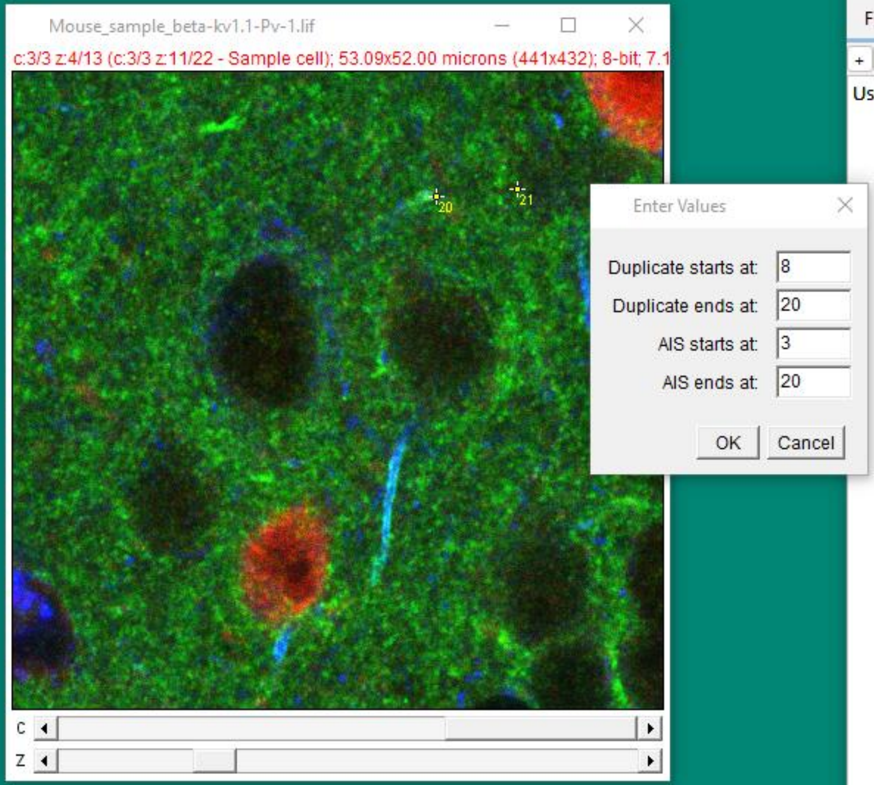

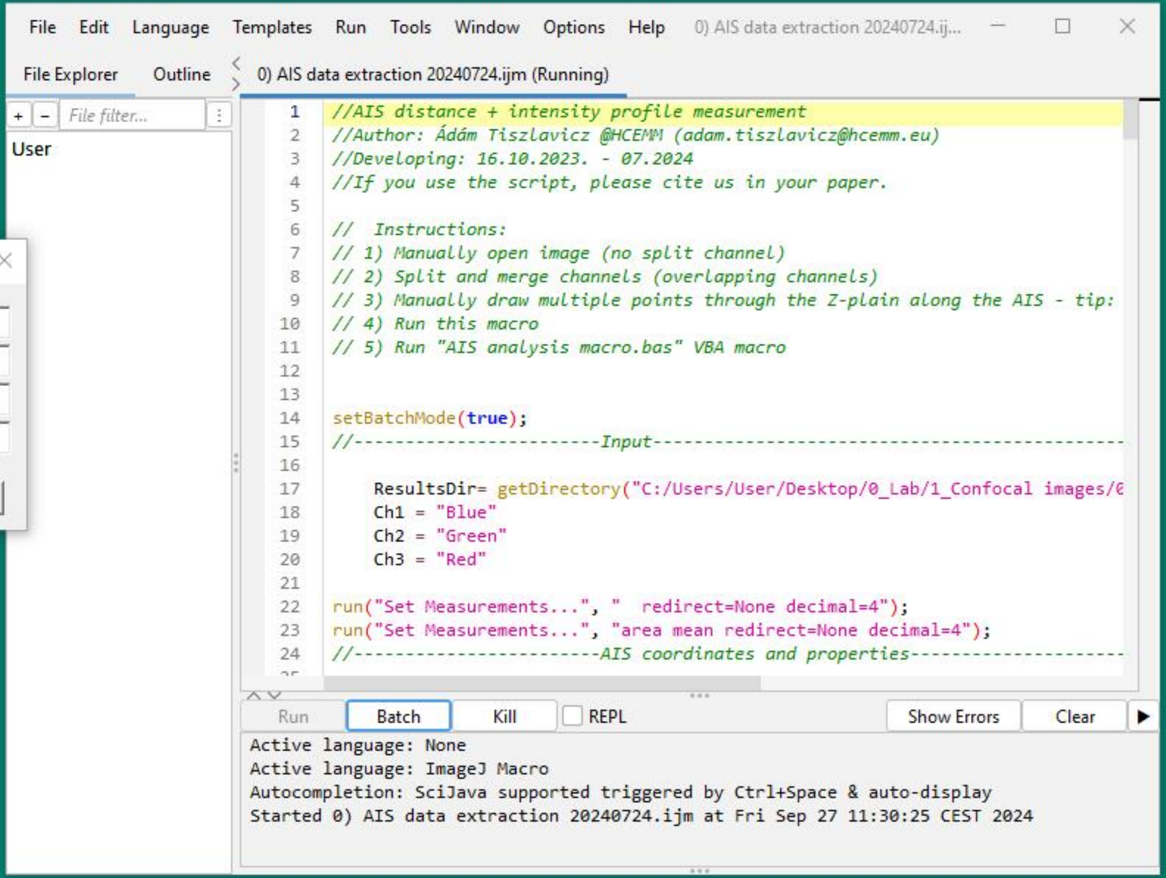

Open and run S8_Code_Excel.bas in Visual Basic (excel)

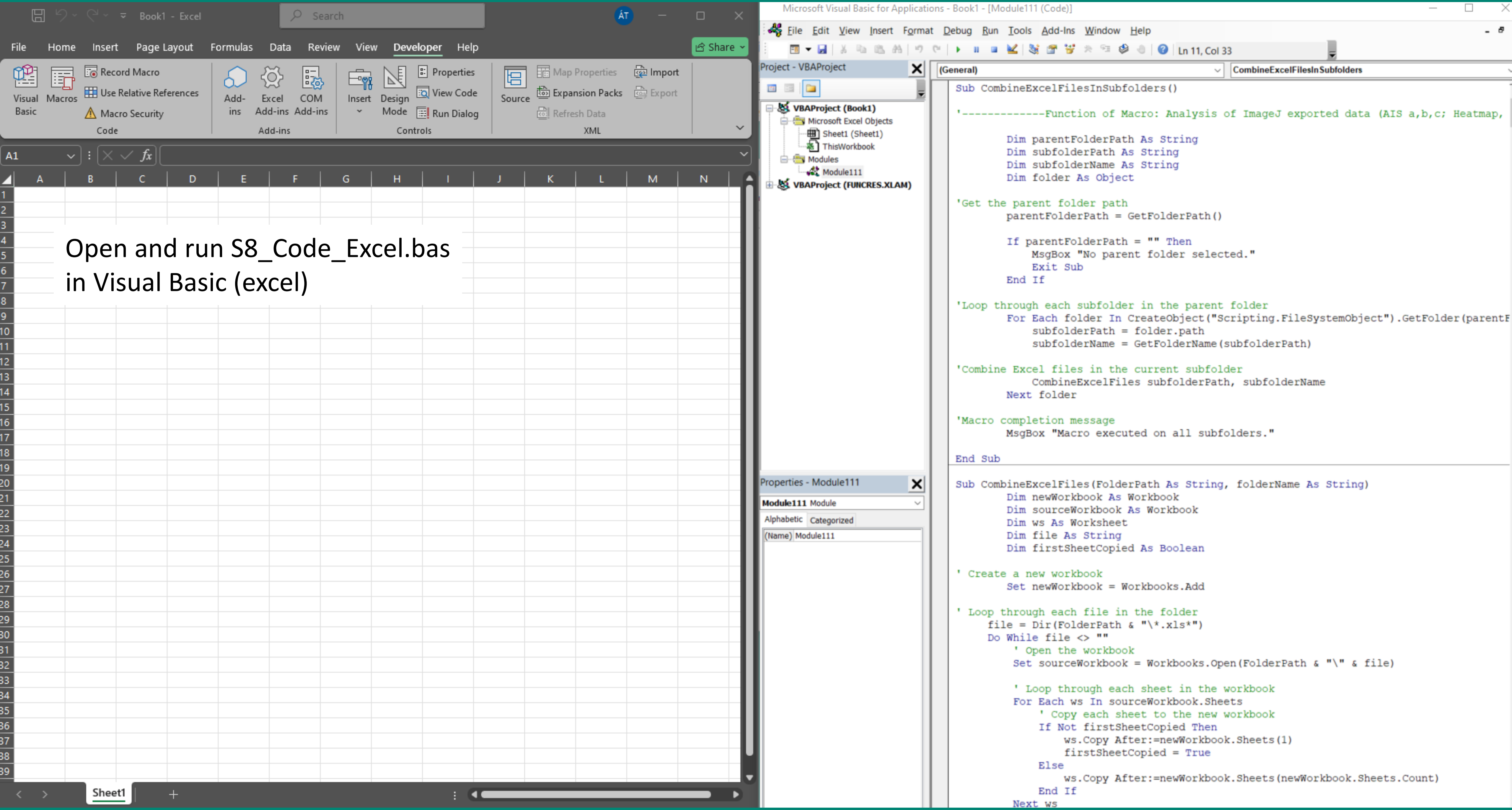

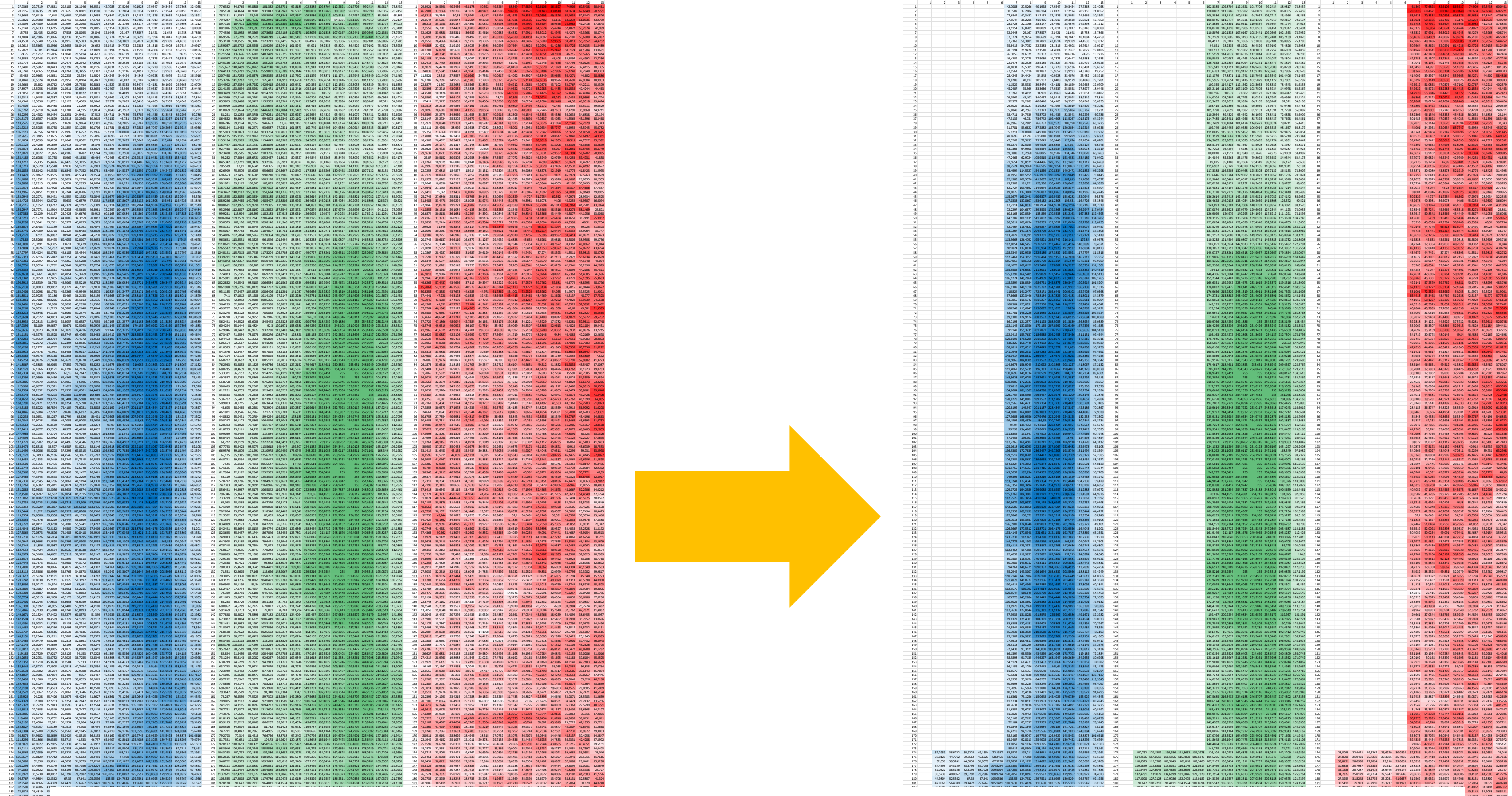

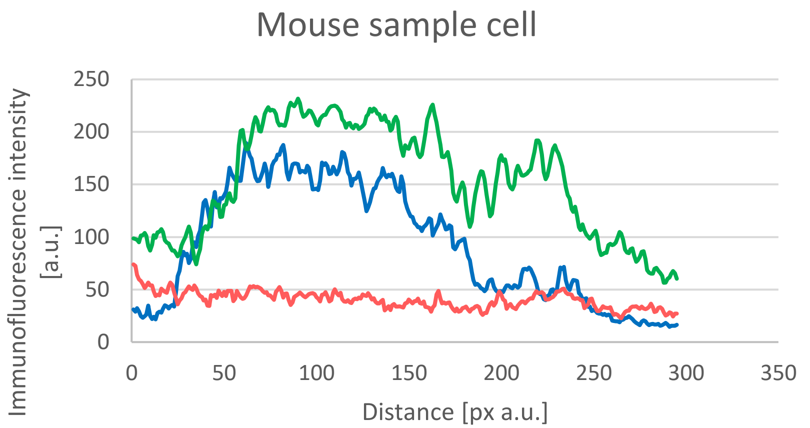

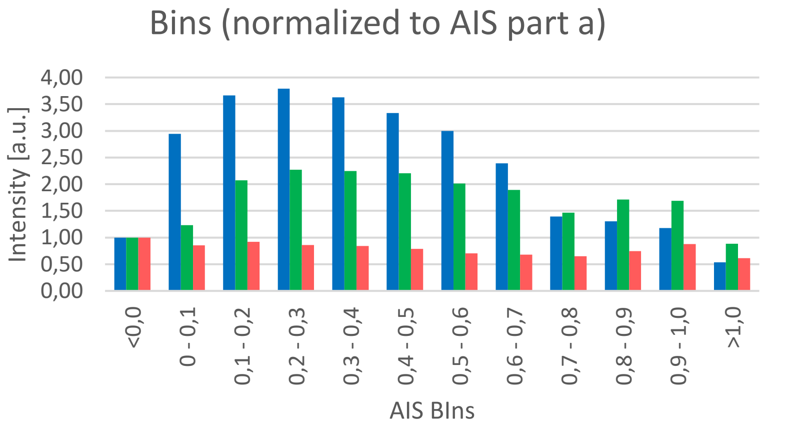

Output excel contains 5 sheets. In abc sheet, AIS proximal distance (a), length (b), distal distance (c) are calculated in µm by using Euclidean distance formula. In sheets Blue, Green and Red, which correspond to channel 1, 2 and 3, heat maps of the AIS are presented. Columns refer to the slice numbers (Z direction) and rows refer to the distance based on the marked points. Heatmaps should be improved by removing values from noise and unrelated signal.

In sheet SUM, averages of the rows and normalization to the background are calculated; and AIS plots are presented.

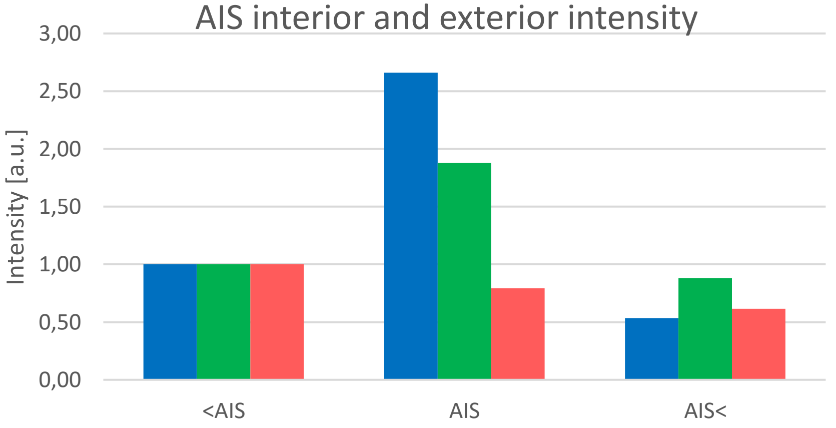

For radial analysis, open image and run S7_Code_ImageJ.jim in ImageJ

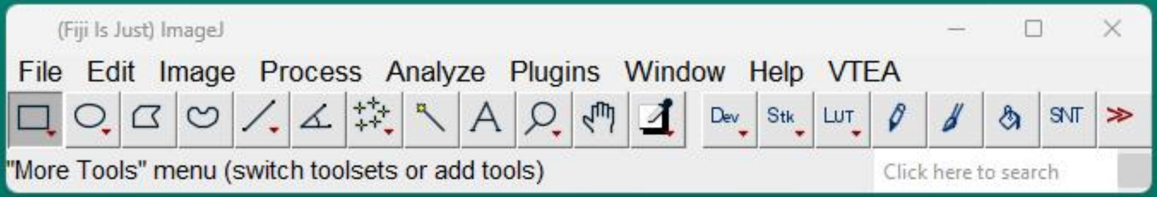

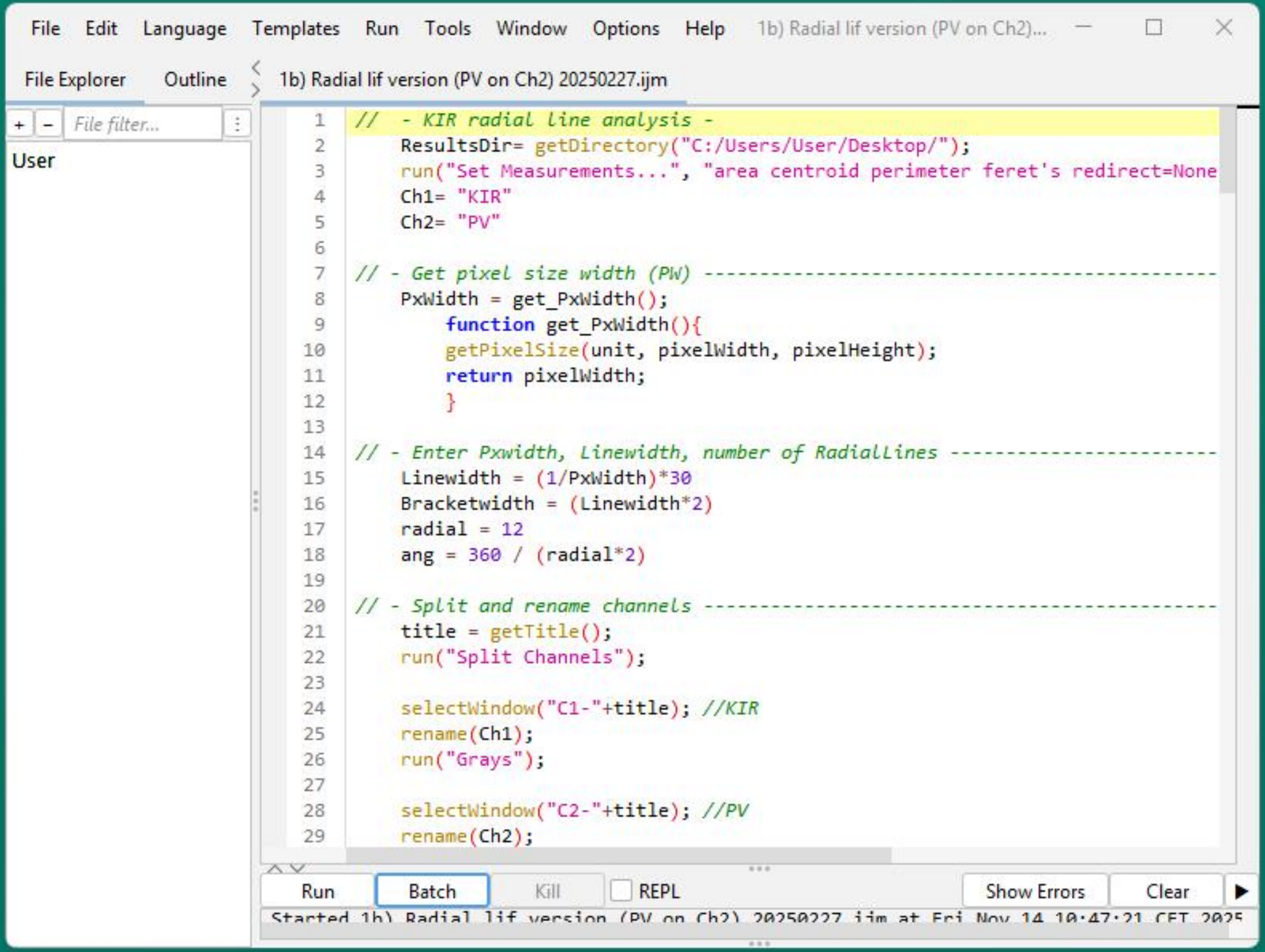

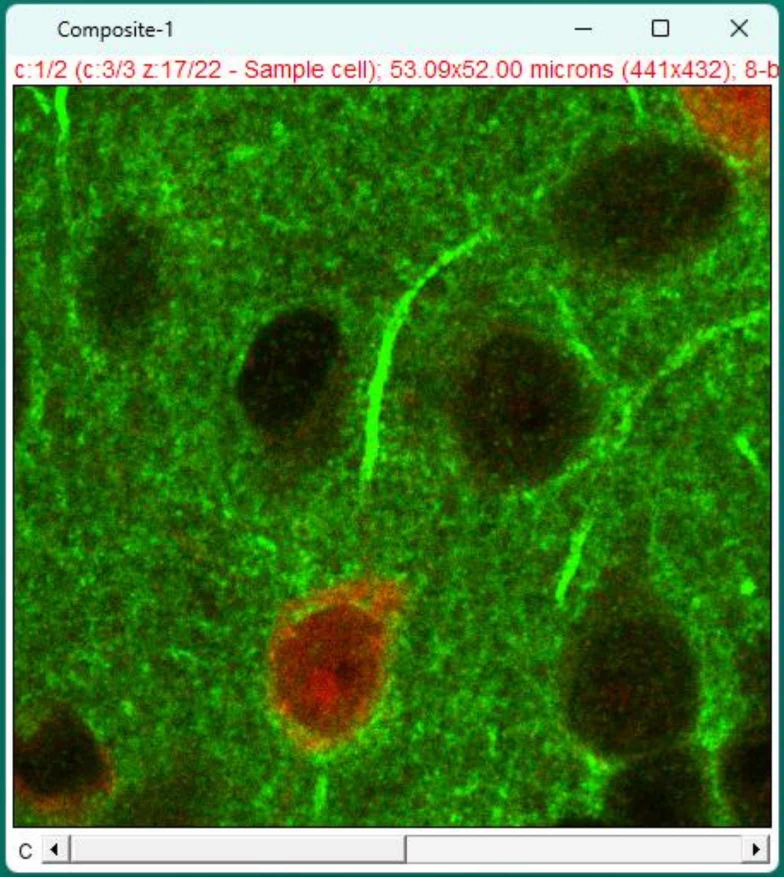

After initiating the script, the code asks for the target folder and selecting soma to be analyzed. The output involves channel images individually and superimposed and data of radial intensity profiles. S9_Code_Excel.bas imports data to excel in which calculations for plotting data can be performed.

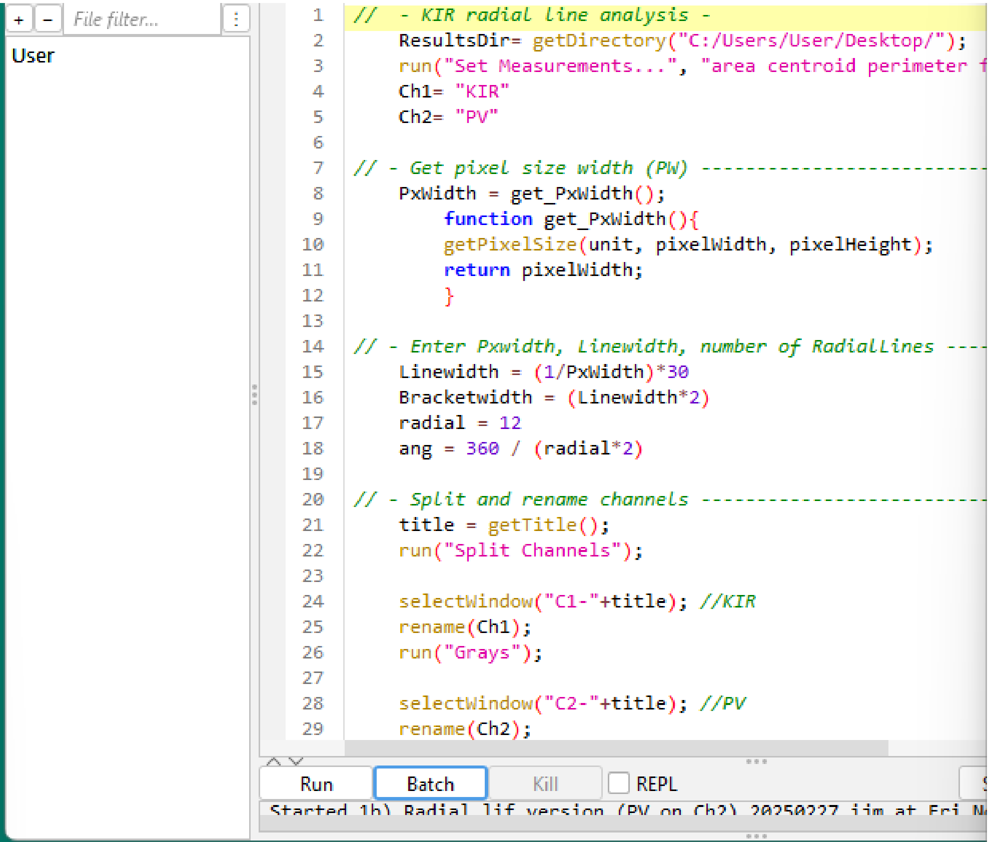

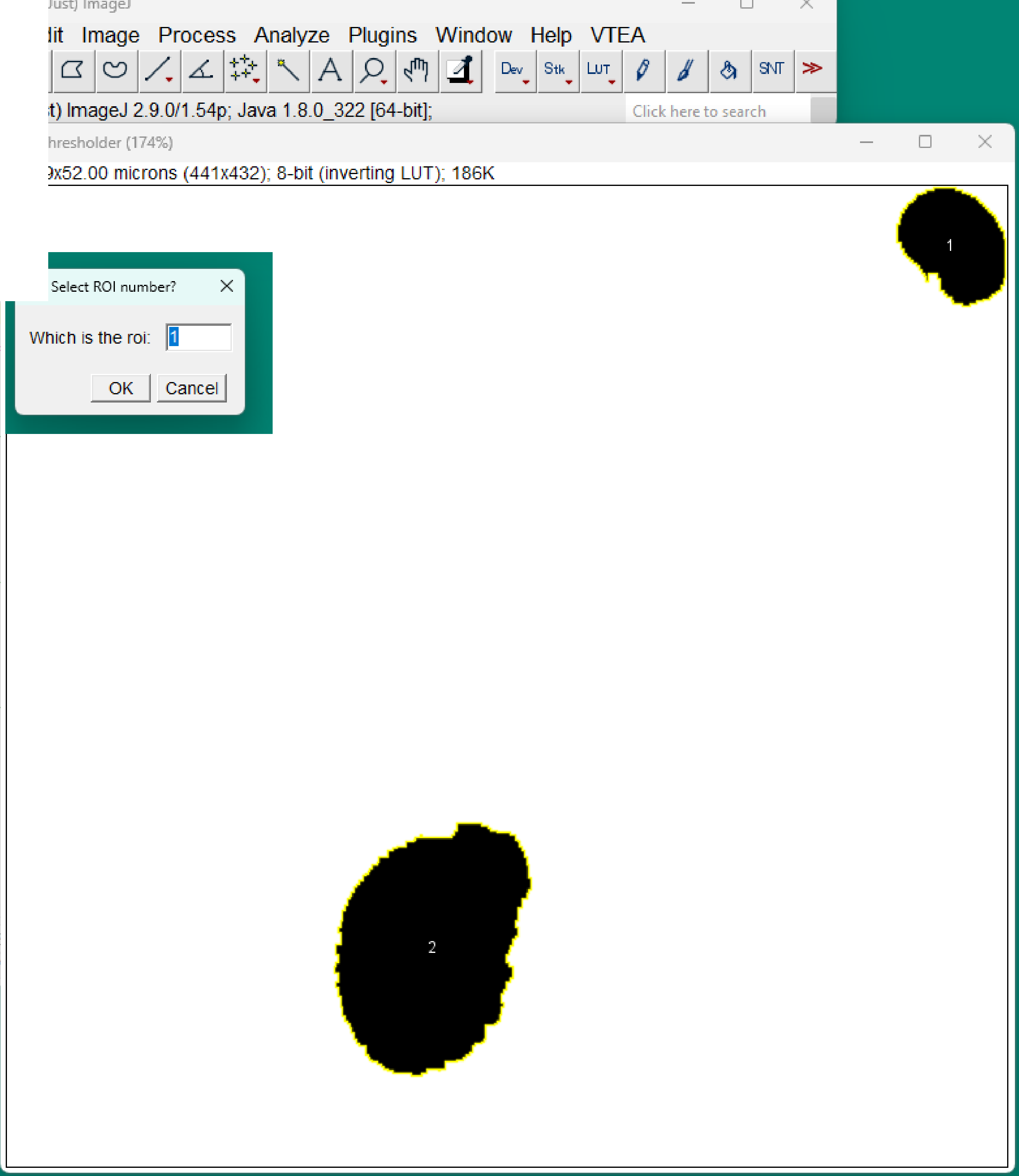

Supplement: S1 Code — (PDF) [file pbio.3003549.s007.pdf]
