# Supplementary material for: Adaptations of the axon initial segment in fast-spiking interneurons of the human neocortex support low action potential thresholds
Source: PLoS Biol. 2025 Dec 10;23(12):e3003549. doi: 10.1371/journal.pbio.3003549 (PMC12798858; doi:10.1371/journal.pbio.3003549)
Supplement: Attachment — Submitted filename: Rebuttal_letter.docx [file pbio.3003549.s012.docx]

*Dear Editors:*

*Please find attached our fully revised manuscript, “Specialized axon initial segment enables low firing threshold and rapid action potential output in fast-spiking interneurons of the human neocortex,” submitted to PLoS Biology. The manuscript has been thoroughly revised in response to the feedback from the editors and expert reviewers. We have also conducted a comprehensive set of new confocal microscopy observations to enable more accurate quantification of subcellular immunofluorescence. Additionally, we included mRNA analysis for other types of ion channels in the patch-sequencing dataset. We added substantial electrophysiological data, including a novel dataset, to demonstrate species-specific differences in neuronal pharmacological sensitivity to the Kv1 channel blocker. These new studies further strengthen our previous conclusions and help resolve inconsistencies between empirical data and the model. We have also added a new simulation parameter to the model in the supplementary data to show the effect of Nav1.6 sodium channel density on the action potential firing threshold in neurons.*

*Please refer to our point-by-point response to the reviewers' comments below for more details.*

*We hope that you and the reviewers will now consider the manuscript suitable for publication in PLoS Biology.*

*Sincerely,*

*Karri Lamsa*

Reviewer #1: This is an excellent study by Bakos and colleagues that provide a detail and thorough analysis of spiking properties of human and mouse interneurons, providing a potential explanation for how human cells maintain fast firing rates despite slower a membrane time constant. The authors effectively demonstrate that the AIS is longer in human cells compared to mouse, but has similar diameters. AP properties are examined thoroughly with very rigorous electrophysiology, showing that AP threshold is lower in human cells. This correlates with a reduction in Kv1 channels in the AIS of human Pv+ cells.

The work is well written and illustrated. I have only a few very minor suggestions that should be addressable with revisions to text or revisions to already existing data/datasets:

1)      The authors should consider expanding the limitations section to note explicitly that immunostaining relies on antibodies that can only be validated by knockout in non-human species.

Alternatively or additionally, an analysis of sequence conservation of mouse vs human Kv1 channels at the antibody binding site would help validate the lack of staining. While it is clear that Pv+ cells largely lack Kv1 labeling in human tissue, there is a possibility that the positively marked initial segment staining is nonspecific. This appears to be more of a concern with Kv1.2 as it appears that there is sub-membranous staining in somata with this antibody.

*-We now explicitly mention this in the limitations section of the discussion. Additionally, we have expanded our immunohistochemical analysis for all three ion channels and added a reference measurement in the extracellular space of each cell. Although this more comprehensive immunoreactivity analysis (new Figures 5–7) does not fully rule out the possibility of false positives, we believe that the conclusions are now more reliable.*

2)      Much of the staining display may be over-saturated, as one cannot discern typical "railroad" patterns for membrane-bound proteins in the AIS. This is always difficult with such small structures, and may be a display issue, but it also relates to my concern about binding specificity. If images could be checked for saturation and corrected if present, that would be very useful.

*-We rechecked all immunoreaction images, including the new immunofluorescence images added to the manuscript, for oversaturation. We apologize for the poor quality of some images in the previous version and hope that their quality is now satisfactory.*

3)      From examples, it appears that human cells have an AP initiation site that is more biophysically separated from the soma in human compared to mouse. This is inferred from the AP phase plane examples in Fig 1 B2 and C2. In human there is an inflection point in the rising phase of the AP that is usually associated with more distal AP initiation. An analysis of the prevalence of these sorts of dynamics could be useful, for example by examining the AIS and somatic components of the rising phase with analysis of dV^2/dt.

*-We have added a comprehensive dataset of action potential (AP) parameters from all human and mouse cells (n = 91 and 95 cells, respectively). The data, including the axon initial segment (AIS) and soma components of the AP (measured as AP phase plot initial rise slope and AP maximum rise speed), are now reported in a new table with between-species comparisons (Table 1). These parameters are also systematically presented in a new set of electrophysiological experiments showing the species-specific effects of dendrotoxin (Kv1 channel blocker) on APs (Table 2).*

*Unfortunately, we could not reliably quantify the phase plot inflection point (which supposedly separates the AIS- and soma-generated Nav components of the AP) as it was absent or barely detectable* *in many cells. We attempted to extract this feature using the second derivative of the phase plot’s rising phase, but this did not yield consistent quantification.*

4)      Just recently, additional leak/stretch sensitive potassium channels have been identified at the AIS in mouse (TREK, TRAAK). Furthermore there are Kv7 channels that are very important for resting Vm and threshold in the AIS. These may be important for subthreshold aspects of excitability in the AIS, perhaps more than Kv1. I fully appreciate that patch seq is both difficult and limited in its interpretation, but if the authors have the data already, an expansion of the display to include all potential potassium-fluxing players in the AIS would be very beneficial.

*-We now report other K-channel mRNA TPM values in the manuscript, with an updated Figure 8 showing TPM heatmaps. We conclude that Kv1 channels are the only K-channel group with lower mRNA levels in human cells compared to mouse cells (c.f. other K channel genes).*

Reviewer #2: In the study conducted by Bakos and colleagues, human parvalbumin-expressing (PV) interneurons from various neocortical regions were compared to mouse PV interneurons. The primary finding indicates that human interneurons exhibit a more hyperpolarized voltage threshold for action potential generation. Geometrical analysis of the axon initial segment, combined with Na+ and K+ channel protein staining, revealed a significantly lower expression of Kv1.1 in humans. Further analysis using a public database and patch-sequencing of the recorded interneurons showed that KCNA1 gene transcripts are significantly lower in humans. The authors continued to develop a computational model to replicate some of the experimental data. The recordings are technically excellent, and the authors gathered an impressive amount of data on parvalbumin interneuron properties in the human neocortex, employing a diverse range of techniques from electrophysiology to single-cell RNA
sequencing, often from the same single interneurons from which functional data were collected. However, this reviewer's enthusiasm for the paper is tempered by several factors: the findings are largely descriptive and correlative. The claim that low Kv1 expression in the human axon initial segment accounts for the lower threshold requires additional experiments, and the role of Kv1 and the axon initial segment needs further substantiation.

1.      Quantifying voltage threshold alone (p. 3) is insufficient to understand the electrophysiological differences between human and mouse interneurons. Kv1 is a fast activating voltage-gated potassium channel known for its role in rapid repolarization and is also slowly inactivating, as described in numerous landmark studies (10.1016/j.neuron.2008.03.003; 10.1016/j.neuron.2012.12.020). Relevant parameters missing include, for example, the delay from current injection onset, firing rates at current threshold (rheobase), current thresholds, action potential amplitude, action potential half-width, afterhyperpolarization, firing adaptation, frequency-dependent width adaptation, etc. Importantly, if the initial segment excitability is the dominant driver in the threshold the components of the action potential phase plane need to be quantified. An integrated view on the physiology is required.

*-We now systematically report additional AP parameters in the revised manuscript. New Table 1 summarizes them, along with between-species statistical tests.*

2.      If the lack of Kv1 at the AIS indeed accounts for a more hyperpolarized voltage threshold in human neurons, pharmacological block should abolish the species-dependent difference. At a minimum, the authors should apply dendrotoxin (Dtx-I, a specific Kv1 channel blocker) to assess its contribution to the action potential parameters (and quantify more than threshold, see #1). Since Kv1 is expressed at both the soma and axon membrane, unlike claims made here in the manuscript, the channel blocker could be applied locally to the initial segment to segregate axonal from somatic Kv1 contribution to membrane excitability. The approach furthermore may show whether other identified differences (e.g. AIS length) contribute (see #3)

*-We performed the dendrotoxin (DTXK) experiments, and the new data are presented in Figure 9 of the revised manuscript. We also detail the drug’s effects on AP parameters in new Table 2.*

3.      Whether the ~3 µm longer axon initial segment plays a role in the action potential threshold is unclear. The correlation analysis is not in line and voltage threshold instead scales with the start distance from the soma (lines 223-235). Computational models deliver only a corollary and cannot be interpreted as conclusive evidence (see point #2). A major concern is that the parameter range is inconsistent with the data; a 10 µm length increase in the model reduces the voltage threshold at the soma by 3 mV (compared to the experimental results putatively showing a ~4 µm more distally positioned initial segment length correlates with no ~10 mV lower threshold). Last but not least, the simulations predict large changes in AP amplitude with lengthening, which are not seen (or not reported) in the data.

*-We agree that the species difference in AIS length is moderate and have revised the manuscript, including the abstract, to emphasize that Kv1 channel deficiency primarily defines the species difference in AP threshold. However, given that the AIS is longer in humans than in mice, we still argue that it likely contributes to the threshold difference. Simulations qualitatively support this: AIS lengthening within the observed range lowers the AP threshold as expected. Regarding the mismatch between empirical data and the computational model, we are working to improve the single-cell model using microanatomical and ion channel localization parameters. Indeed, we have initiated new collaboration with Prof. Michele Giugliano to develop a more accurate model in the future.*

4.      There are major concerns about the language used for the electrophysiology. A few examples, 'firing threshold' is a vague term. Voltage- and current thresholds are absolute measures but depend on many technical confounding factors including membrane potentials and duration of the current injections.

*-We now explain these concepts more clearly in the main text (at first mention) and in the table legends (e.g., AP threshold for a square-pulse current step applied at the soma, hereafter referred to as “firing threshold”).*

Furthermore, voltage-gated channels are not 'inhibitory' (e.g. line 59 and elsewhere) since that is used to describe the impact of synaptic conductance.

*-We clarify this in the text as follows: “Kv1 channels are clustered in the AIS and soma in rodent pvalb neurons, where their presence as low-threshold inhibitory channels raises the AP firing threshold (hereafter, Kv1 channels are referred to as inhibitory channels).”*

Also "Kv activity" is unclear and ambiguous (line 381). Are the authors referring to channel opening, or perhaps channel conductance densities?

*-We now define this in the text as conductance at first mention and refer to the figure and figure legends, where Kv and Nav activity is modeled as conductance per μm^2^.*

Other issues are noted at line 98 (and throughout the entire manuscript) where the authors write "electrical resistance". Neurons have an input resistance, reflecting the local cell's membrane response upon current injections, encompassing aspects of geometry as well as the intracellular and membrane resistances, in units of Ohm. Membrane resistance, however, is a unity measure, referring to the specific feature of the membrane (Ohm cm2).

*-We now use these terms more precisely, referring to input resistance when discussing directly measured resistance values in the cell.*
At p. 9 they write about "real-life pvalb neurons", departing from scientific language. Please rigorously edit the manuscript an expert to make the accessible to expert and broader audience.

*-We now use “real” in most places, leaving “real-life” only in two instances where it more loosely refers to cells in genuine brain tissue.*

5.      The strongest correlation was found between the membrane time constant and the voltage threshold (line 180). Lines 492-500 are meant to provide an explanation, but incomprehensible and extremely speculative without having data on the morphology or the passive membrane properties (all voltage-gated conductances being blocked). Time constant also depends on the channel composition. The unexplained time constant requires pharmacological approaches

*-With this plot and correlation, we aimed to demonstrate the tight association between the actual speed (measured as time constant) at which depolarizing membrane potentials are generated and the AP firing threshold. We agree that “time constant” or “tau” here reflects not only passive membrane properties but also various voltage-activated components. However, we consider pharmacological and molecular analysis of input resistance and tau beyond the scope of this study. We now define the “time constant” as the apparent time constant and explain what it represents in this context.*

Reviewer #3: Bakos et al. report the results of an interesting study assessing differences between parvalbumin-positive (PV) neuron in slices from human and mouse neocortex. Given that data from human PV cells are very scarce, the comparison done in this paper could be very useful for many colleagues in the neuroscience community. There are several points that the authors should carefully address before the study can be acceptable for publication. Please, see my comments below.

Major

1) The study compares AP threshold across species using the definition: "the Vm at which the AP initial rise speed reaches 10 mV/ms (Fig. 1A1)." However, the differences between mouse and human cells could vary depending on how AP threshold is defined. Since these differences are central to the paper's conclusions, the authors should clarify why this specific definition was chosen. Would the human-mouse differences change if the threshold were defined using 20 or 5 mV/ms instead of 10 mV/ms?

*-The threshold value of 10 mV/ms is consistent with values reported in previous literature for fast-spiking interneurons in the neocortex of humans and rodents (Wilbers et al. 2023), monkey and rodents (Povysheva et al. 2008), and mice (Li et al. 2014), as cited in our manuscript. We also tested alternative thresholds of 5 and 20 mV/ms in a random sample of human and mouse cells. However, this had negligible impact on the firing threshold, as the steep onset of the AP results in minimal shifting along the abscissa for such changes in the ordinate (as is seen in Fig 1B3 and 1C3).*

2) As shown in Fig. 2B, the authors measured the membrane time constant using depolarizing current steps. This approach is problematic because depolarizing steps often activate subthreshold voltage-gated channels (below the AP threshold). Since the membrane time constant is a passive property, it should be measured under conditions that avoid activating voltage-gated channels. Typically, this is achieved using small hyperpolarizing current steps, which minimize such activation.
Given that the authors have measured multiple membrane properties, such as input resistance and capacitance, their dataset likely includes responses to hyperpolarizing current steps, which are essential for accurately estimating the membrane time constant in the hyperpolarizing range. This information should be incorporated into the manuscript to ensure a proper and "uncontaminated" analysis of the time constant. Otherwise, the between-species comparison may be confounded by using a method that measures time constants affected by voltage-gated channel activation. Indeed, Fig. 2B1 suggests that the depolarizing steps in human neurons (but not in mice) produce a voltage "hump," indicative of voltage-gated channel activation. Hyperpolarizing steps could of course activate hyperpolarization-activated currents (Ih) mediated by HCN channels, which are widely expressed and have been reported in human PV neurons in published studies. However, HCN is sometimes considered part of the leak channels that regulate input resistance (Rin) and tau near resting membrane potentials, hence HCN channel activation is expected when measuring these passive properties. In contrast, depolarization activates a wide array of voltage-gated channels, which can significantly skew measures of Rin and tau.

*-We used depolarizing voltage steps for tau measurement for two reasons:*

*1) In this study, tau specifically reflects the kinetics of membrane depolarization toward the AP threshold, which is central to the main focus of the work: how excitatory input dynamics influence AP generation.*

*2) In certain neuron types (including pvlab neurons in the neocortex) hyperpolarizing membrane potential (Vm) steps are affected by strong Kir3.1 and Kir3.2 channel conductance in the somatic membrane (Goldberg et al. 2011, cited in the manuscript). As a result, tau and input resistance (Rin) for hyperpolarizing steps are typically faster (tau) and smaller (Rin) than those for depolarizing pulses in the same cell. Additionally, tau measurement for hyperpolarizing pulses in these cells is difficult to define because Kir conductance becomes progressively more activated at increasingly hyperpolarized Vm levels, i.e., tau and Rin are highly dependent on the degree of hyperpolarization. Therefore, hyperpolarizing steps in human pvalb neurons do not serve as stable, passive time constant measures, unlike in some other neuron types. This has been shown in mouse neurons (Goldberg et al. 2011, DOI: 10.1093/cercor/bhq138). In a 2024 study, we also characterized human and mouse Rin and tau properties across Vm values (Mechanisms and purpose of lowered action potential firing threshold in fast-spiking interneurons in the human neocortex. E Bakos, A Tiszlavicz, V Szegedi, A Douida, S Furdan, D Welter, J Landry, ….bioRxiv, 2024.10. 28.620622).*

3) Lines 174-177: Regarding point 2 above, the lack of correlation between Rin and tau in human neurons, as well as the similar tau values in cells with high and low Rin, is unexpected. Tau is typically proportional to the product of membrane resistance and capacitance (Tau = Rm * Cm), so tau and Rin are generally correlated, with higher Rin associated with higher tau values.
One possible explanation for this discrepancy is that the membrane time constant was estimated using depolarizing steps. As shown in Fig. 2B1, the depolarizing steps in human neurons appear to be "contaminated" by the activation of voltage-gated channels, which could disrupt the expected correlation between these passive properties. To resolve this, it is essential to measure Rin and tau using small hyperpolarizing current steps, minimizing voltage-gated channel activation. This would confirm whether the observed human-mouse differences are due to genuine differences in passive membrane properties or the differential activation of voltage-gated channels.

*-We agree that lack of correlation in human neurons (p = 0.085) is notable and likely reflects considerable cell-to-cell variability in membrane conductance (both passive voltage-activated) across the population of human pvalb neurons. This variability in conductance per unit area influences Rin between the resting potential and AP firing threshold. Therefore, we interpret these results to mean that similarly sized human pvalb cells (with similar capacitance) can still display markedly different Rin and tau, likely due to differences in leak conductance per membrane area (nS/μm^2^). Accordingly we measured tau and Rin from the same depolarizing Vm steps, as shown in Fig. 2A1 and 2B1.*

*Again, we emphasize that our study focuses on membrane properties between the resting potential and AP firing threshold, making this Vm range most relevant. Additionally, as noted earlier, pvalb neurons differ from many other neuron types (except, for example, some thalamic principal cells), as their Rin and tau decrease substantially and progressively with hyperpolarizing Vm (Goldberg et al, 2011; Furdan et al. 2025).*

4) The experiments examining AIS properties (Fig. 3) are very elegant, and I commend the authors for this impressive work. However, I am somewhat unclear about whether the authors propose that the actual AP initiation site is located specifically within this anatomically defined axonal compartment (as identified by beta-spectrin labeling) and how this relates to the differences in AP threshold.
In particular, the interpretation of the correlations in Fig. 3C is not entirely clear. The strongest correlation is between AP threshold (APthr) and AISa (p = 0.012). Does this suggest that AP initiation occurs in this segment of the axon? By contrast, the correlation with AISb—the AIS segment supposedly well-defined by beta-spectrin labeling—is not significant. This discrepancy raises questions about the precise role of these AIS regions in AP initiation.

*-Notably, this dataset is small (measuring AP threshold first, followed by analyzing successful triple immunoreaction and intact AIS) and shows substantial cell-to-cell variability. Consequently, these outcomes likely reflect random variability and differ somewhat from our larger datasets on AP threshold and AIS anatomy, which were collected independently. However, we included these results because they suggest that AIS positioning along the axon is associated with the AP firing threshold, although the correlation is less clear in this smaller dataset compared with the larger, independent dataset. Our purpose in using biocytin-filled cells was to assess both electrophysiological (AP threshold) and anatomical (AIS features) parameters in the same human neurons, rather than comparing independent samples. However, both AP threshold and AIS anatomy (e.g., distance to soma and length) showed considerably cell-to-cell variability. The data imply that moderate sample sizes are insufficient to draw strong statistical conclusions about parameter associations.*

I believe the interpretation of these results requires more careful consideration in the manuscript.

*-We now state explicitly that “**The large cell-to-cell variability of AIS length and localization was also evident in the correlation between AP threshold and AIS structure within the same cells: due to the variability in both measures, the results remained less clear and partially diverged from the correlation findings in the larger, independent datasets.”*

Additionally, the implications for the spatial location of the AP initiation site should be discussed in greater depth. It would be valuable to reference and compare these findings with published studies that have directly recorded from the axon to assess the AP initiation site, including cases where axonal AP thresholds were measured directly, even if not in PV cells from the human cortex. This would provide a broader context and strengthen the discussion.

*-We elaborated the original text further, noting that “**Although this work does not determine the exact AP initiation site in the AIS, it shows that the low-threshold ion channels Nav1.6 and Kv1 are, on average, evenly distributed along the AIS, without a consistent intensity peak at either end.”*

*We performed new analyses quantifying ion channel immunofluorescence signals along the AIS in individual cells (intensity profile along the AIS) and across species (human vs. mouse cells) with statistical testing included. These results confirm that all three low-voltage ion channels show a smooth distribution along the AIS, without evidence for a peak (e.g., for Nav1.6 channel intensity) at either end. Between-species immunofluorescence quantification for the ion channels is based on our new analysis, referencing each cell’s signal (for ~240 cells with ion channel staining in the AIS) to its own extracellular calibration region (which is average of approximately 3,000 control reference pixels per cell). AIS measurements used a density of ~50 pixels/μm, providing a robust reference.*

5) The rationale for investigating the relationship between AP threshold and axon diameter or Nav1.6 distribution needs clarification.

*-We now explain this rationale in the text as follows: “**We examined axon diameter in biocytin-filled pvalb interneurons to assess its potential correlation with the AP firing threshold, particularly across species, as axon caliber can influence axon excitability (48).” Although we found that axon diameter does not explain the species-specific difference in AP threshold, it may still contribute to cell-to-cell variability in threshold within each species. We illustrate the complex relationship between axon diameter and AP threshold using simulations in the revised Figure S1.*

While axon diameter is important for subthreshold voltage propagation, with active membrane properties the situation could be quite different. The implications of Nav1.6 distribution patterns or gradients along the AIS (proximal to distal) should also be better explained.

*-We performed new study quantifying AIS Nav1.6 immunofluorescence, as noted in our response to point 4. The new analysis (revised Figure 5) tested whether Nav1.6 signal intensity peaks at the first or second half of the AIS proximal–distal axis. Results revealed even distribution along the axon in human pvalb neurons. This pattern was already known for rodent pvalb cells (Li et al. 2024, cited in our manuscript).*

It is unclear how the reported AP threshold differences between human and mouse neurons relate to specific Nav1.6 density gradients or distributions, and clarifying these connections would help.

*-The new analysis presented in Figure 5 enables a more confident comparison (with extracellular ca. 3000 pixel intensity reference in each cell for the pixels in the AIS) of Nav1.6 immunofluorescence signal intensity between species. The results show no significant difference in Nav1.6 signal intensity in the AIS between humans and mice. These findings are now discussed in the manuscript.*

Lastly, species differences in AP thresholds might reflect variations in Nav1.6 density, but immunohistochemistry has limitations for comparing channel densities across species due to antibody variability and labeling efficiency. Acknowledging these issues and suggesting alternative methods would strengthen the discussion.

*-We now specifically mention this point in the limitations paragraph in the discussion. However, we improved the reliability of intensity comparisons between species by systematically referencing fluorescence signals (i.e., F-Fexc) as we already mentioned above. As we now explain in the manuscript, every* *pixel measured for ion channel immunoreaction was compared to the average intensity of ~3,000 pixels measured separately in the extracellular space from the same cell’s local environment. Identical referencing was used for Kv1.1 and Kv1.2 immunoreactions (new Figures 6 and 7).*

6) The immunohistochemistry/mRNA data for Kv1 channels suggest that human PV neurons have very low levels of Kv1.1 in the AIS membrane. However, the quantification method may exaggerate species differences.

*-We now illustrate identical analysis of mRNA TPM for same genes (including Kv1.1) using the Allen Institute open access dataset and their pvalb cell subtype classification system based on mRNA transcriptomics. The Allen dataset (showing average TMP per identified pvalb cell type) similarly shows that Kv1.1. levels are significantly lower (systematically undetectable) in human cells compared with mouse cells.*

In Fig. 6A and B, data are normalized to pre-AIS signal levels, assuming low beta-spectrin levels pre- and post-AIS. It's unclear if Kv1 channel density truly differs across these compartments, *making species comparisons with this normalization approach challenging.*

*-We have now performed analysis using new and comprehensive datasets, as described earlier in response to point 5. This analysis, based on extracellular reference average intensity for each cell, allows more reliable estimation of ion channel density intensity in subcellular loci. Results revealed that although no species difference was found for Kv1.1 signal intensity at the pre-AIS or post-AIS level, a robust intensity difference was observed specifically in the AIS.*

For example, it's hard to conclude that Kv1.2 levels in the AIS are low; Fig. 6B5 mainly shows that these levels remain constant relative to pre-AIS, with an F/Fpre-AIS ratio near 1. Both Fig. 6A and B suggest Kv1 levels are low relative to beta-spectrin in the AIS of both species, but more so in humans.

*-We applied similar new experiments and signal analysis to Kv1.2 immunoreactions (new Figure 7).*

By the same reasoning, one might infer that PV levels are lowest in the AIS. However, Fig. 6 does not provide enough evidence to determine if PV levels in the AIS are low compared to other AIS proteins or across species. The patch-seq data might clarify some of these uncertainties, though I have reservations, as discussed in point 7.

*-We have not included pvalb immunoreaction quantification in the AIS, but we retained signal analysis of pvalb as a binary marker confirming the presence or absence of pvalb positivity.*

7) Including patch-seq data, as in Fig. 7, could provide highly valuable insights, but the presentation of the data raises some questions. For instance, if the blue color in the heatmap represents TMP values close to zero, it suggests that nearly half of the neurons recorded in human and mouse cortical slices lack detectable PV mRNA.

*-For clarification, we now present mRNA TPM analysis of pvalb neocortical neuron subtypes, as classified by the Allen Institute based on neuronal mRNA transcriptomics, in new Figure 8. In humans, many pvalb cell subtypes exhibit markedly low pvalb gene expression levels (blue colors). For further clarification, we have marked the actual pvalb neuron subtypes, using specific identity codes shown on the abscissa, in both our patch-sequencing dataset and the Allen Institute dataset. To our knowledge, it remains unclear why pvalb mRNA levels are so low in many human pvalb cell types. One possibility is that protein turnover is so slow that transcription levels are low under the conditions in which tissue was resected during surgery.*

*Our patch-sequencing dataset confirms the low pvalb gene expression in the human cells, as we argue that the mRNA analysis genuinely reflects low or undetectable levels (human cells had a high number of active genes: 6,784 genes, IQR = 4,872–7,399, n = 21 cells; mice had fewer genes: 3,617 genes, IQR = 3,173–4,305, n = 16).*

Were the cells included in the patch-seq experiments confirmed to have PV protein immunoreactivity? This could be challenging, as extracting the nucleus for patch-seq might disrupt the neuronal soma, complicating immunodetection of PV protein.

*-Cells included in the patch-sequencing experiments were not further studied via immunohistochemistry (they were electrophysiologically studied prior to nucleus harvesting).*

If all cells in the Fig. 7A1 heatmap were confirmed PV protein-positive, the absence of detectable PV mRNA in ~half of these neurons would indicate that PV mRNA is not uniformly detected in PV neurons from human and mouse cortices. This raises concerns about interpreting patch-seq results, as the absence of PV mRNA—the primary marker for this cell type—suggests that RNA expression profiles should be analyzed with added caution.

*-In the Figure 8, we now present the Allen Institute database analysis of average TPM levels for genes (including PVALB) in parallel with patch-sequencing heatmaps.*

Similarly, SLC6A1, which encodes the vesicular GABA transporter GAT1, a marker for all interneuron subtypes, is detected in only ~half of human neurons. This further complicates the interpretation of patch-seq data.

*-We argue that the observed result could be explained simply by markedly low expression levels of the gene in human cells. Notably, although our mRNA analysis, performed and supervised by the EMBL GeneCore facility and Dr. Vladimir Benes, reveals transcribed gene levels, the protocol we currently use may be less sensitive than the Allen Institute’s protocol for detecting genes expressed.*

Additionally, the statement, "mRNA analysis of patch-sequenced human and mouse pvalb neurons (upper schematic illustrates the protocol) revealed the absence of KCNA1 expression in all human neurons, whereas it was robustly expressed in many mouse cells," seems inaccurate. According to the heatmap, KCNA1 mRNA is undetectable in 10 of the 16 mouse PV cells, with most other cells showing only low expression levels. This discrepancy should be addressed to ensure accurate representation of the data.

*-We now support this statement by presenting analysis of the Allen Institute open dataset analysis in Figure 8, alongside our patch-sequencing data.*

8) While I greatly value computational modeling data, in this study, it would be crucial to demonstrate that at least some of the findings from the model are predictive of experimental differences in spiking activity and firing patterns between PV cells in mouse and human cortices. Including experimental data comparing spike trains would significantly enhance the manuscript, as some readers may perceive the scope as too narrow.

*-We have now generated a completely new dataset to investigate the effects of Kv1 channel blockade on AP properties in human and mouse cells. The results show (as predicted by the model) that the mouse AP threshold becomes similar to the human AP threshold. We did not systematically quantify other AP parameters in the modeling study (which involved multiple AP features), as replicating them exactly from real-cell pharmacological experiments would be challenging. For instance, modulation affected AP maximum rise and fall speeds differently, likely because Kv1 level adjustment in the model more strongly influenced Nav1.6 activation in the AIS (explaining the increased maximum rise speed and consequently greater Kv3-mediated afterhyperpolarization) compared with the effect in real cells. Specifically, Kv1.1 blockade in real cells mainly reduced maximum fall speed, possibly because somatic Kv1 channels contribute to more afterhyperpolarization in real neurons relative to their effects in the model.*

Specifically, the observed mouse-human differences in AP properties likely have important consequences for input-output transformations in neurons. This could be addressed in model neurons by varying the amount and temporal patterns of excitatory current.

*-This is an exciting idea. In the model, we use a range of input strengths to observe EPSP-spike transformation, as illustrated in new Figure 10F3. However, for the remainder of the simulation (Figure 10G), which examines the EPSP-spike lag relationship, we use a single EPSC conductance level to maintain focus (as these simulations already include two variables, AIS length and gKV1, with multiple EPSC intensities; Figure 10F3).*

*Additionally, the authors could conduct current-clamp experiments in slices to test the model's predictions regarding firing pattern differences between mouse and human PV cells.*

*-This is also an exciting idea, and we plan to continue testing it in the model and real cells in future studies. However, in the present study, which already includes ten main figures, three tables, and one supplementary figure, we consider this aspect beyond the current scope.*

Minor

- Lines 146-156: the authors compare AP threshold between human frontal and temporal cortex slices, but it is not clear if AP threshold differs in comparison with any other areas, nor what are was recorded in slices from mouse cortex. Please clarify.

*There was no difference in recordings from human tissue in other cortical areas compared with the temporal or frontal cortex (MANOVA and Mann–Whitney U test pairwise comparisons with Bonferroni correction). Likewise, no difference was observed between mouse cells from somatosensory versus frontal cortex (Mann–Whitney U test).*

- Lines 137-138, please state what area of mouse neocortex was studied.

*-We apologize for the omission of the n-numbers in the original manuscript. These are now included in the text. Mouse cells were studied in the somatosensory cortex (n = 45 cells) and frontal cortex (n = 50 cells).*
- Lines 140-141: "to identify the lowest threshold that triggered APs" 
This statement sounds odd. It appears that a cell can have multiple AP thresholds and the authors somehow to pick the lowest of all. Cell could have different thresholds to trigger an AP, but if so, this happens in different membrane compartments, like soma versus mid-dendrite versus distal dendrite. Since the experiments in this study do not involve recordings from different compartments, the sentence is not clear.

*-We agree with this comment, and the sentence has been rephrased accordingly (i.e., we removed the word “lowest”).*

- Lines 155-156: "The results showed that human pvalb neurons have a lower AP firing threshold potential in response to excitation in the soma than mouse pvalb neurons." 
While this is true, the authors should explain under what type of excitation the differences were observed (current steps/pulses?).

*-The AP induction protocol is now clarified in the text as follows: ‘We applied positive Vm steps (square-pulse current steps of* *increasing amplitude, 500 ms at 1 Hz) to cells current-clamped at −70 mV to identify the AP threshold (Fig. 1B, C) (defined as the Vm at which the AP initial rise speed reached 10 mV/ms; hereafter, referred to as the firing threshold; Fig. 1A1).*

- Lines 159-161:  The text would be clearer if the cortical area is indicated.

*-This is described in an earlier paragraph: “We measured AP firing thresholds in 91 pvalb-expressing fast-spiking interneurons (43) from human neocortical tissue resected during deep brain surgery in 67 patients (Table S1). The cells were studied using whole-cell clamp recordings in layer 2/3 of acute brain slices sectioned from the frontal (n = 39 cells), temporal (n = 25 cells), and parietal, occipital or other neocortical regions(n = 27 cells) from male (n = 30) and female (n = 34) patients aged 11–85 years.”*
- Lines 162-164: "We measured the average Rin and Cm in neurons in Vm steps of +5 to +10 mV (elicited by square pulse currents), which were excitatory but below the AP firing threshold (subthreshold) when applied at −70 mV (Fig. 2A1)." It is more appropriate to say that the current pulses were depolarizing, since depolarizing steps that are subthreshold can have a mix of excitatory and inhibitory effects.

*-Although the reviewer is correct,* *we would prefer to retain this sentence in its original form. For clarity and accessibility to the journal’s broad readership, we aim to generalize at this point in the text.*
- Lines 210-213: "from 28 human and 22 mouse cells recorded in the whole-cell clamp, the intact soma and full-length AIS were successfully recovered (demonstrated by beta-IV-spectrin immunofluorescence) in 13 human and 14 mouse neurons." I may be misunderstanding this sentence, but the numbers of cells with recovered axon appear low, given that, typically, when FS interneurons are filled with biocytin recovery of large fractions of axon is common (usually all the axon that "survived" slicing). Hence, finding axonal labeling beyond the AIS should be relatively common and achieved in many recorded cells, even if the full complement of axon branches is not recovered.

*-It is true that recovering biocytin-filled cell partial axon is straightforward. However, further confirmation steps were essential: double immunofluorescence reaction had to be successful for the pvalb and betaIVspectrin antibodies, and the axon needed to initiate from the soma with a fully visualized AIS. A complete AIS was confirmed if biocytin-filled axon continued after beta-IV-spectrin labelled AIS (illustrated in Fig. 3B1). Thus, we only included cells meeting all these criteria. The reported yield reflects this selection. These cells were electrophysiologically characterized prior to microscopy.*

I am wondering if this sentence actually refers to cells in which beta-IV-spectrin immunofluorescence was successful?

*-The reported numbers include all cells that were electrophysiologically recorded and subsequently visualized via biocytin staining. Failed cells include cells with ruptured or damaged soma, failure to confirm continuation of the axon after the betaIVspectrin-labelled AIS, or failure to measure AIS parameters clearly because of unsuccessful immunoreaction in human tissue.*

If recovery of the axon is much lower in FS cells recorded in human cortex slices, compared to mice, then this should be clearly stated.

*-We did not note any such difference between the species. Human tissue had additional challenges in betaIVspectrin immunoreactions.*
-Fig 1A2-3: is it not clear if the analysis of PV+ axon segments in A3 was done in the reconstructed PV neuron shown in A2

*-This was the case, and it is now clearly stated in the revised text.*
