# Supplementary material for: Adaptations of the axon initial segment in fast-spiking interneurons of the human neocortex support low action potential thresholds
Source: PLoS Biol. 2025 Dec 10;23(12):e3003549. doi: 10.1371/journal.pbio.3003549 (PMC12798858; doi:10.1371/journal.pbio.3003549)
Supplement: Attachment — Submitted filename: Response to Reviewers_210925.docx [file pbio.3003549.s013.docx]

Dear Editor and Reviewers,

Thank you for your comments and constructive feedback on our research manuscript. We have addressed the issues you raised and have provided a point-by-point response below.

In our response to Reviewer 2's first major comment, we discuss additional AP properties besides the firing threshold and rheobase current. You may find this discussion in the manuscript.

Regarding Reviewer 2's second major comment, we conducted a thorough statistical analysis of experiments involving human and mouse pvalb neurons exposed to the Kv1.1 receptor blocker DTXK. This new analysis comprises a mixed-design ANOVA and post hoc tests with a p-value adjustment to account for familywise errors. The analysis shows how the twelve parameters responded to DTXK exposure in both human and mouse neurons. Importantly, the analysis indicates that DTXK altered the two key features, the AP threshold and the rheobase current, differently in human and mouse neurons. The full analysis of human and mouse cells, including an increased n-number from new experiments, is presented in updated Table 2 and Figure 9, which detail the statistical tests.

Regarding Reviewer 2's third major point, we revised all statements and conclusions concerning the role of AIS length in the action potential (AP) threshold difference between human and mouse neurons. We acknowledge that AIS length likely contributes to the large variability observed in AP threshold within species. We discuss this as follows: "Although AIS length contributes to AP threshold variability among individual pvalb cells in both species, it does not explain the interspecies difference. While an increased AIS length increases the number of Na⁺ channels in the AIS, making it easier to reach the AP threshold (as demonstrated in Supplementary Figure 1), an elongated AIS does not necessarily generate differences in AP thresholds between species." The spike threshold is influenced by various factors, including axial resistance between the soma and AIS, as well as electrical resistance within the AIS. Possible species differences in these parameters may mask the effect of moderately elongated AIS on AP thresholds in human neurons.

You will also find our response to Reviewer 3's comments in the rebuttal letter below. We corrected some typos in the manuscript and the labeling in Figures 1 and 10.

The following updated files have been uploaded: Figure 9; the manuscript text with the new Table 2 (clean text versions and a version with highlighted changes). We uploaded metadata files for Figure 9, Table 2, and Figure 8 (showing the mRNA TPM values).

We would like to thank the editor and reviewers again for carefully reading the manuscript and providing constructive comments that helped us improve it.

We hope you find the manuscript suitable for publication in PLOS Biology.

Yours,

Karri Lamsa

------------------------------------

REVIEWS:

**Reviewer #2:** In this revised manuscript, the authors have undertaken several new experiments, which is commendable and has the potential to enhance the overall quality of the work. However, in addressing the concerns raised by this reviewer, the rebuttal and the interpretation of the new data appear to be inconsistent with the findings. My principal concern remains that the manuscript does not provide direct evidence to support its central claim—that the properties of the "specialized axon initial segment" (AIS) account for the observed threshold differences.

**Major Point 1:** The authors have now quantified a greater number of parameters, as presented in the updated Table 1. Nevertheless, neither the rebuttal nor the manuscript offers a clear interpretation or contextualization of these data. For instance, what is the significance of the observed increase in half-width duration? Similarly, how should the differences in latency be understood?

In the manuscript, we now discuss the AP properties to provide context.

We state in the Results: “They (human cells) also exhibited a lower rheobase current…, which is the amplitude of current needed to reach the AP threshold, compared to mouse cells”. We state on the discussion: “Due to the lower AP threshold and higher input resistance, human cells require less current to reach the spike threshold”.

We explain meaning of the AP initial slope: “Human cells showed a moderately lower AP initial rise slope…. This parameter determines the angle of the tangent line fitted to the early rise of the AP waveform. It measures the AP component generated in the AIS.”.

In addition, we explain that human cells had a slower AP maximum fall speed and a wider AP half width: “Human cells had a slower maximum fall speed than mouse cells. However, the maximum rise speed of APs was similar across species, and no differences were observed in AP peak values. These results demonstrate that the AP repolarization phase, mediated by an active K⁺ current, is faster in mice than in human neurons. Consequently, it is logical that human neurons also have a wider AP half-width.”

We explain meaning of the finding on AP generation delay difference between the species: “Compared to mouse neurons, human cells exhibited shorter latency to AP generation at rheobase (p < 0.001), suggesting that they have specific mechanisms that facilitate AP firing and shorten the AP lag.”

Additionally, we briefly discuss the role of Kv1.1 and the DTXK effect on AP properties, cell input resistance, and membrane time constant (tau) in human versus mouse cells (see our response to the point 2 below). We mention that Kv1 channels appear to be partially responsible for the lower input resistance and faster tau observed in mouse cells compared to human cells when depolarizing membrane potential steps are measured. Thus, Kv1 channels regulate not only the AP threshold potential, but also somatic excitability through input resistance and tau.

**Major Point 2:** The original concern pertained to whether differences in Kv1 expression at the AIS are sufficient to explain the electrophysiological properties observed. The authors have conducted new experiments involving the selective Kv1.1 blocker dendrotoxin-k (DTX-k), as shown in the new Table 2 and Figure 9. While these experiments are valuable, the rebuttal fails to articulate the insights gained from them and draws erroneous conclusions. A key issue lies in the statistical analysis of the data presented in Table 2 and Figure 9. The authors compare DTX-k versus baseline within each species and then statistically assess "differences of differences" in Figure 9, which is methodologically flawed. To properly evaluate whether Kv1 differential expression accounts for the distinct threshold and other action potential parameters, a two-way repeated measures analysis of variance is required. Subsequent post-hoc comparisons of the absolute data should then be used to determine whether the means of the action potential parameters in the presence of the blocker are comparable between human and mouse neurons.

The data and analysis in the updated Table 2 and Figure 9 are now analyzed using a mixed-design ANOVA and post hoc tests with Holm's correction, which adjusts p-values to control the family-wise error rate. We acknowledge Dr. Fanni Nedenyi, of the HCEMM Scientific Computing Core Facility's Modeling and Statistics Unit, for her expert assistance with the analysis.

The mixed-design ANOVA indicates a significant interaction between species (human vs. mouse) and time (baseline vs. 10 minutes of DTXK treatment) on the measured values for AP threshold (AP Thr). This means that the effect of the DTXK treatment on AP Thr over ten minutes differs between mouse and human species. Post hoc tests confirm that both species show a significant change in measurement values from baseline to 10 minutes after DTXK, but the magnitude of this change appears larger in mice. Additionally, there is a significant difference in baseline measurements between species: humans have more negative baseline AP Thr values. However, this difference is no longer significant after 10 minutes of DTXK treatment.

For rheobase current, the DTXK has a significant effect within the mouse data but not the human data. Rheobase values differ between species at both time points (in baseline as well as in DTXK). We summarize these results now in the manuscript as follows: “Comparisons of the effect of DTXK across species revealed that, in addition to a AP threshold shift, the rheobase current was affected differently in mice than in humans (mixed-design ANOVA interaction, p = 0.0082). The rheobase current was reduced in mice (p = 0.0015), but not in humans (p = 0.4648; post hoc Wilcoxon tests with Holm correction) (Fig. 9C1-3)”.

Table 2 and Figure 9 have been updated to show the results of the mixed-design ANOVA and post hoc tests. Post hoc tests were applied when the mixed-design ANOVA test indicated significant differences in either between-species (comparing pooled human versus pooled mouse data of both time points), between-time-points (comparing pooled data of both species between time points), or interaction effect (time-related changes differed between species). Wilcoxon signed-rank post hoc tests were performed within each species, and Mann–Whitney U tests were performed between species at each time point. Holm-adjusted p-values were used for multiple comparisons.

We discuss the significant effects of DTXK by the analyses on other parameters besides AP Thr and rheobase current and explain how these alterations can be attributed to Kv1 channel blockade.

In the Results section, we state the following: "Although the rest of the observed parameters failed to reveal a significant difference in the DTXK effect between humans and mice (mixed-design ANOVA interaction, p > 0.05), Kv1 channel blockade was associated with a reduction in the amplitude of the action potential afterhyperpolarization (AP AHP) in mice, but not in human cells. This difference reflects the stronger somatic Kv1 channel contribution to AP AHP in mice compared to human cells in control conditions. The AP half width, which was shorter in mice under baseline conditions, did not remain significantly shorter after the wash-in of DTXK, reflecting the blockade of K^+^ current components of the AP in mice. Additionally, DTXK was associated with a similar reduction in the maximum positive peak value of the AP in both species. This suggests that the effect of DTXK on the AP occurred through a mechanism other than direct Kv1.1 channel current. (A mixed-design ANOVA test with post hoc pairwise comparisons are shown in full in Table 2.).

In addition, we continue in the Results section that: “DTXK significantly increased input resistance in mouse cells but not in human cells. Kv1.1 channels are activated in the cell soma by Vm depolarization, which reduces Rin under baseline control conditions. Blocking these channels with DTXK increased the input resistance. Consistent with this finding, DTXK reduced species differences in membrane tau. Tau differed more between species in control conditions than in the presence of DTXK (see Table 2). The results indicate stronger Kv1 channel activity in the AIS and soma of mouse neurons compared to human pvalb neurons."

**Major Point 3:** The authors assert that "given that the AIS is longer in humans than in mice, we still argue that it likely contributes to the threshold difference." However, no evidence is provided to substantiate the claim that AIS length directly influences voltage threshold. In fact, the data in Table 2 suggest that, in the presence of DTX-k, action potential thresholds are similar across species, despite the longer AIS in humans. This implies that the modest length difference of approximately 3 µm does not contribute to a lower threshold. This conclusion is further supported by the authors' own immunofluorescence analysis (Figure 6), which demonstrates that Kv1 channels are not exclusively localized to the AIS membrane but there is also a lower fluorescence detectable at the somatic membrane.

Indeed, a global reduction in KCNA1 expression and Kv1 protein levels would also affect somatic membrane properties, potentially explaining the strong correlations observed with

somatic rise times.

We rewrote all the statements and revised the conclusions regarding the AIS length to explain the difference in AP thresholds between humans and mice. These changes are highlighted in the revised manuscript.

We state in the manuscript discussion: "Although AIS length contributes to AP threshold variability among individual pvalb cells in both species, it does not explain the interspecies difference. While an increased AIS length increases the number of Na⁺ channels in the AIS, making it easier to reach the AP threshold, as demonstrated in Supplementary Figure 1, an elongated AIS does not necessarily generate differences in AP thresholds between species. The spike threshold is influenced by various factors, including axial resistance between the soma and AIS and electrical resistance within the AIS. Possible species differences in these parameters may mask the effect of moderately elongated AIS on AP thresholds in human neurons.”

We state in the manuscript Results for Fig 3 that “Because the number of cells studied electrophysiologically and investigated anatomically is relatively small, and because the range of measured AP threshold (AP Thr) and AIS structure values is large, it is possible that our dataset is not large enough to reveal hidden correlations between AIS geometry and AP threshold within each species. Nevertheless, these findings suggest that the difference in AP threshold between human and mouse pvalb neurons cannot be explained by AIS length or axonal location. However, they suggest that the position of the AIS may partly explain the large variability observed in the AP threshold between individual neurons. Specifically, a more distal AIS placement is associated with a lower AP threshold”.

**Major Point 3, Continued:** The authors acknowledge that the neuronal model lacks sufficient detail and have initiated a new collaboration with Dr Giugliano to address this. However, it is unclear how a future study would resolve the fundamental concerns raised in the current manuscript, particularly given the aforementioned arguments that AIS length differences alone do not play a significant role.

In the computational modeling section of the study, we state that the two types of AIS used in the simulation resemble human AIS (with no Kv1 channels) and mouse AIS (with high Kv1 channels), but they also have AIS lengths that are either elongated or shortened. We explain that the two types of AIS were used in the AP lag experiments to maximize the AP Thr difference when testing the association between AP Thr and time lag to AP generation.

Importantly we conclude in the simulation study that: “Overall, these simulations demonstrate that lower AP firing thresholds, generated by AIS parameters that differ between human and mouse real pvalb neurons, are accompanied by shorter time lags to AP generation in response to synaptic currents at the soma. Importantly, the simulations show that variability in Kv1 content alone in the AIS is sufficient to regulate AP threshold (ordinate values in Fig 10D) and EPSP onset-to-AP lag (ordinate values in Fig 10G)”.

The figure 10 legend has been changed, and we now state the following: “Switching the model's AIS properties from mouse-like (high Kv1 content) to human-like (no Kv1) while simultaneous changing the AIS length from short to long reduced the AP generation lag to somatic excitation.”

**Reviewer #3:** I thank the authors for their detailed response to my previous comments. I think the revised manuscript will be significantly improved. Before the manuscript is ready for publication, there are still a few points that need to be addressed. Please, see below.

1) In their responses to my first comment: "We used depolarizing voltage steps for tau measurement for two reasons: …" the authors justify why depolarizing steps were used, characterizing special conditions inconsistent with the "traditional" meaning of Tau, the membrane time constant of a neuron. While the authors' approach could be, in principle, OK, they need to clearly specify in the text of the manuscript (to avoid confusing future readers, especially non-expert readers of PLoS Biology) that the meaning of tau in their manuscript is different from the consensus tau. While the functional meaning of the time constant of a neuron is partly ambiguous (see for instance, A brief history of time (constants), C Koch, M Rapp, I Segev 1996, PMID: 8670642), the authors should clarify that their measurement named as "tau" diverges from the meaning of the membrane time constant "tau" measured under standard conditions (see, for instance, Spruston and Johnston 1993, PMID: 1578242).

The membrane property typically named "tau" relates to the passive membrane property. While tau is obviously not a purely passive property in any real neuron, is quite different from the measurement that the authors name "tau" in this manuscript. Simply changing the name used in the manuscript and specifying why the conditions of the measurements do not assess the standard "tau" would be sufficient.

We now refer to it as "tau" (with quotation marks) throughout the manuscript and define this when the term is first mentioned in the Results section as apparent tau i.e. “tau”. We also define "tau" as the apparent membrane time constant in the Table 2 legend. In the Methods section, we explain it as follows: "The membrane potential time constant, or apparent tau (“tau”), was measured from the rising phase of 5-10 mV depolarizing steps generated by 250-500 ms square-pulse positive current steps in a physiological solution without drugs."

2) The responses to my second comment: "…Again, we emphasize that our study focuses on membrane properties between the resting potential and AP firing threshold …", still suggest the risk of confusing future readers. Indeed, the trajectory of the membrane potential between resting and the threshold voltage to trigger a spike is a very important and interesting property to study and to compare between PV interneurons across species. But this does not mean that the time constant measured by the authors could be named "tau" without stating some qualifier upfront. As remarked in my previous comment, the authors need to make revisions to make clear to future readers that the time constant they have measured most likely does not reflect "tau" in the standard/consensus meaning of the word.

We have addressed this important point systematically in the manuscript (see our response to point 1 above).

3) The authors response: "moderate sample sizes are insufficient to draw strong statistical conclusions about parameter associations" seems reasonable, but the revised text of the manuscript is weak/vague in this regard. It should be revised to make sure the future readers clearly understand which conclusions are not possible to make from the authors' data, because of the small sample size and large cell-to-cell variability.

*With this statement, we refer to the whole-cell experiments and post hoc anatomical analysis of cells that we performed in parallel in human and mouse cells.* *Rather than vaguely stating it in the discussion, we now explain it as a conclusion in the Results section after the experiments, as follows:: “Because the number of cells studied electrophysiologically and investigated anatomically is relatively small, and because the range of measured AP threshold (AP Thr) and AIS structure values is large, it is possible that our dataset is not large enough to reveal hidden associations between AIS geometry and AP threshold within each species. Nevertheless, these findings suggest that the difference in AP threshold between human and mouse pvalb neurons cannot be explained by AIS length or axonal location. However, they suggest that the position of the AIS may partly explain the large variability observed in the AP threshold between individual neurons. Specifically, a more distal AIS placement is associated with a lower AP threshold.”*

4) I don't think the authors properly responded my question of why they propose that axon diameter is crucially relevant for active propagation of signals (mediated in part by Nav1.6 channels) … Their response states some immunohistochemistry findings related to the subcellular distribution of Nav1.6 immunofluorescence with the AIS … : "We performed new study quantifying AIS Nav1.6 immunofluorescence, as noted in our response to point 4. The new analysis (revised Figure 5) tested whether Nav1.6 signal intensity peaks at the first or second half of the AIS proximal-distal axis…" But how and why does this matter in terms of role of diameter in signal propagation through the AIS? Please clarify the sentence/s.

We now explain this in the revised manuscript's Results section, where we introduce the experiments for measuring axon diameter.

“Thicker axons have lower longitudinal resistance (Ra) in the AIS. This allows for faster passive electrical conduction between the soma and the AIS. However, the lowered resistance in the axon simultaneously increases the AIS's AP rheobase current. However, larger axons also have more ion channels, including voltage-gated sodium channels that initiate the action potential, suggesting a complex relationship between axon diameter and AP Thr.”

In the modeling part of the study and in the supplementary Fig S1, we further elaborate on the complex theoretical relationship between AP Thr and axon diameter. These simulation experiments demonstrate that variation in axon caliber in a range of measured parameters in real cells can change the AP Thr, but its effect remains small. This is because an increased diameter results in a lowered Ra and an increased number of Na⁺ channels, which move the AP Thr (for currents applied in the soma) in the opposite direction. We mention this in the Results section of the manuscript (highlighted in the revised manuscript). We also summarize this in the discussion section as follows: “Increasing AIS length reduces AP threshold more effectively than expanding axon diameter, although both increase the number of AIS ion channels. However, expanding axon diameter also lowers Ra, which shifts AP threshold in the opposite direction and limits its effect”.

5) The complexity in interpreting the patch-seq data (PVALB and SLC6A1 expression levels, among others) highlights the possibility that the quality of the patch-seq data was not sufficient to properly support the claims made by the authors. This should be more strongly emphasized in the revised manuscript.

*In the Results section, after reporting the patch-sequencing data, we added the following sentence to explain possible reasons for the occasional undetectable levels of mRNA for some genes in our dataset of individual neurons: "Natural fluctuations in gene expression levels in individual cells may explain the occasional undetectable mRNA in our patch-sequenced cells. However, it is also possible that our system occasionally loses mRNA or misses its conversion* to cDNA for reasons that are not yet understood."

Additionally, we now specifically mention in Figure 8 legend that "0-level indicates that the mRNA expression level is below the detection threshold."

Also, please see our response to point 6 below.

6) The authors state: "although our mRNA analysis, performed and supervised by the EMBL GeneCore facility and Dr. Vladimir Benes, reveals transcribed gene levels, the protocol we currently use may be less sensitive than the Allen Institute's protocol for detecting genes expressed". While this situation is understandable, the statement highlights the limitations in comparing/aligning the patch-seq data from the present study with the Allen Institute databases. This limitation should be clearly mentioned in the revised manuscript, ideally including a brief technical explanation of why the protocol employed in their study is less sensitive.

We now explain this in the manuscript text.

In the Results section for Figure 8 data, we now mention the following: "The heatmaps based on our patch-sequencing data and the Allen Institute data differ in that our dataset shows raw TPM results in individual neurons, whereas the Allen Institute data show the average TPM level for multiple cells of that cell type. This may explain why, unlike the Allen Institute dataset, ours occasionally shows undetectable mRNA levels for common genes, such as SLC6A1, in some cells. It is also noteworthy that the Allen Institute dataset shows an absence of mRNA levels for PVALB in some cell types, even though the dataset represents the average of many cells.”

We use the same method as the Allen Institute for sample mRNA collection and sequencing. Additionally, we only include cells in which we identify mRNA for a significant number of genes. As we mention in the Results section, we detected mRNA for 6,784 genes in humans and 3,617 in mice (median), which highlights the quality of our dataset. Note that the Allen Institute reports the trimmed mean expression of up to 100 or more cells in each subclass to represent the genes. "Trimmed mean" means that data below the 25th percentile and above the 75th percentile are excluded to calculate the average value shown in the heatmaps. Thus, both very small and very large values are removed within each subclass of cells. Consequently, their data never shows occasional zero expression for genes such as gene A or gene B, whereas our single-cell representation does.

We also report the real TPM values of the patch-sequencing data heatmap in “Fig 8 Metadata” excel file available for any further analysis by readers.

Although we are not certain why our dataset occasionally shows zero levels for some common genes, we would like to suggest three possible explanations.

Possibility 1: Gene expression in mammalian cells is often bursty and stochastic rather than continuous, and mRNA TPM levels can vary 100-fold between transcription bursts and silent periods (Robles-Rebollo et al., Nature Communications 13, 4342 [2022]. https://doi.org/10.1038/s41467-022-31192-9; Tukacs et al., bioRxiv 2025. https://doi.org/10.1101/2024.05.16.594495). This natural fluctuation of individual gene expression rates can introduce noise into the overall dataset. Although the phenomenon is well recognized in fields such as cancer research and molecular biology, it is not emphasized in neuroscience. This feature may also partly explain the occasional weak quantitative correlation between mRNA and protein.

Possibility 2: The mRNA level is too low for our method to detect it. However, we consider it unlikely that the mRNA would not be detected with 4 million reads. We emphasize that we only accept cells with a high number of active genes, which is a standard for high-quality patch sequencing. Furthermore, it is noteworthy that some cells with a very high number of active genes, e.g., 8,500, show a level of 0 for the GAT-1 or PVALB mRNA.

Possibility 3: Some mRNA has degraded, or the cDNA conversion was unsuccessful for that particular mRNA. This would mean that, for reasons unknown to us, our system might lose mRNA or fail to convert it to cDNA for some specific genes. However, we stress that we have no evidence supporting this possibility. It is possible that some mRNA degradation occurs in some samples, leading to the disappearance of mRNA from genes that are already naturally present in low amounts in the sample. We also mention the latter possibility in the manuscript.

7) Regarding my minor comments about recovered AIS beta-IV-spectrin immunoreactivity etc., the statements made by the authors in their responses should be incorporated in the text of the manuscript, either in methods, in results or both sections, depending on the specific point.

In the Results section, we added the following: "The total number of cells includes all cells that were electrophysiologically recorded and subsequently visualized via biocytin staining. Cells that failed include pvalb cells with ruptured or damaged somata, failure to confirm continuation of the axon after the beta-IV-spectrin-labeled AIS, and failure to clearly measure AIS parameters due to an unsuccessful immunoreaction in human tissue. The double immunofluorescence reaction had to be successful for the pvalb and beta-IV-spectrin antibodies, and the axon had to initiate from the soma with a fully visualized AIS. A complete AIS was confirmed if the biocytin-filled axon continued beyond the beta-IV-spectrin-labeled AIS (Fig 3B1).”
