# Supplementary material for: Adaptations of the axon initial segment in fast-spiking interneurons of the human neocortex support low action potential thresholds
Source: PLoS Biol. 2025 Dec 10;23(12):e3003549. doi: 10.1371/journal.pbio.3003549 (PMC12798858; doi:10.1371/journal.pbio.3003549)
Supplement: Attachment — Submitted filename: Reviewer comments.docx [file pbio.3003549.s014.docx]

Please see our detailed, point-by-point responses below.

**Title:
-- We would like to suggest a tweak of your title to streamline it and emphasize the main findings. Does this alternative formulation work for you?

"Adaptations of the axon initial segment in fast-spiking interneurons of the human neocortex support low action potential thresholds"

Thank you for this suggestion. We agree that the new title works well, so we have changed it.

**Financial disclosure statement:

-- Please add links to the funding agencies in the Financial Disclosure statement in the manuscript details.

-Links to the funding bodies were added to the manuscript.

**Ethics:  
-- Thank you for including information about your study's ethical approvals. As your ethics statement does not explicitly mention the mouse work, please confirm if these approvals covered both the human and animal work, and add the necessary information if not.

-We added this in the text.

-- For animal work, we require the full name of the IACUC/ethics committee that reviewed and approved the animal care and use protocol/permit/project license. Please also include an approval number, and the specific national or international regulations/guidelines to which your animal care and use protocol adhered. Please note that institutional or accreditation organization guidelines (such as AAALAC) do not meet this requirement.

-The full name of the ethics committee that reviewed and approved the animal work, along with the permit number, is now mentioned in the text. Additionally, the international guidelines to which the animal work adheres are mentioned.

-- Please make the Ethics Statement a separate, independent (and the first) subheading in the Materials & Methods section.

-Done

Please separate the license information into a different sub-heading.

-Done.
 
**Data and Data Availability:

-- We noticed that your data availability statement in the submission form and in the text do not precisely match (in the form, you say that all data are available without restriction, but in the text, you say that underlying data and code are available upon request). While we appreciate that you have included the data underlying the figures in the supplement, we strongly encourage you to make all publicly available and to bring these statements into alignment.

-The data availability statement in the text is now in line with the submission form.

-- Again, thank you for providing the data underlying the figures in the supplement. However, we would like to request that you re-name these files to match our standard format. Please make sure that all data files are uploaded as 'Supporting Information' and are referred to invariably using the following format verbatim: S1 Data, S2 Data, etc. in the file names and descriptions throughout your manuscript. For example, you can rename the file currently titled "Metadata Figs 1-4 New.xlsx" as "S1_Data.xlsx".

-All files were renamed as suggested in both the text and on the submission website, where the files were uploaded as supporting information.

-- Related to this, please add descriptions of the supplemental data files to the manuscript, and please include in the figure legends where the underlying data can be found, e.g. “The data underlying this Figure can be found in S1 Data”

-The sentence has now been added to the figure and table legends.

 **Code availability:

-- Per journal policy, if you have generated any custom code during the course of this investigation, please make it available without restrictions. Please ensure that the code is sufficiently well documented and reusable, and that your Data Statement in the Editorial Manager submission system accurately describes where your code can be found.

-We added custom code files and instructions for using the code in the ImageJ program and Excel. These files were uploaded as S5_Code Instructions, S6-S7_Code ImageJ, and S8-S9_Code Excel. We also added a sentence to the Methods section stating that the custom codes and instructions for using them are available in S5_Code Instructions, S6-S7_Code ImageJ, and S8-S9_Code Excel files.

-- Please note that we cannot accept sole deposition of code in GitHub, as this could be changed after publication. However, you can archive this version of your publicly available GitHub code to Zenodo. Once you do this, it will generate a DOI number, which you will need to provide in the Data Accessibility Statement (you are welcome to also provide the GitHub access information).

-We provide DOI number of the manuscript file in a repository.

Journal Requirements:

- We do not publish any copyright or trademark symbols that usually accompany proprietary names, eg ©, ®, ™ (e.g. next to drug or reagent names). Please remove all instances of trademark/copyright symbols throughout the text, including:

™ on page 47.

-These have now been removed from the text (on page 47), as advised.
